# Exploring the impact of flow dynamics on corrosive biofilms under simulated deep-sea high-pressure conditions using bio-electrochemostasis

**DOI:** 10.3389/fmicb.2025.1540664

**Published:** 2025-02-28

**Authors:** Nicolò Ivanovich, Enrico Marsili, Xinhui Shen, Elena Messinese, Marcos, Pauliina Rajala, Federico M. Lauro

**Affiliations:** ^1^Singapore Centre for Environmental Life Sciences Engineering, Nanyang Technological University, Singapore, Singapore; ^2^Nottingham Ningbo China Beacons of Excellence Research and Innovation Institute, University of Nottingham, Ningbo, China; ^3^School of Mechanical and Aerospace Engineering, Nanyang Technological University, Singapore, Singapore; ^4^Department of Chemistry, Materials and Chemical Engineering “G. Natta”, Politecnico di Milano, Milan, Italy; ^5^The UK Foreign, Commonwealth and Development Office, London, United Kingdom; ^6^Asian School of the Environment, Nanyang Technological University, Singapore, Singapore; ^7^Nanyang Environment & Water Research Institute (NEWRI), Nanyang Technological University, Singapore, Singapore

**Keywords:** high-hydrostatic pressure, microbially-influenced corrosion, sulfate-reducing bacteria, chemostat, biofilm

## Abstract

The formation of biofilms on metal surfaces contributes to the degradation of metallic materials through a process known as microbially influenced corrosion (MIC). While MIC accounts for a substantial portion of the global corrosion-related costs, its study is particularly challenging when related to infrastructure deployed in extreme environments inhabited by microorganisms, such as the deep sea. Here, this limitation was addressed with the development of a high-pressure bio-electrochemostat able to simulate the conditions of the deep sea more accurately than the traditional closed-batch setups. With this device, the corrosive capabilities of the piezophilic sulfate-reducing bacterium (SRB) *Pseudodesulfovibrio profundus* were analyzed at 0.1 (atmospheric pressure) and 30 MPa under flow and static conditions on AH36 marine-grade carbon steel. The results highlighted the device’s ability to closely replicate environmental conditions, thereby keeping bacterial communities metabolically active throughout the experiments and allowing for a more accurate assessment of the impact of MIC. Furthermore, the comparison between atmospheric and high hydrostatic pressures clearly showed that MIC represents a threat for metallic structures at the bottom of the ocean as much as at surface level.

## 1 Introduction

Microbially influenced corrosion (MIC) is the process in which the degradation of a material, typically a metal, is driven by the presence and metabolic activity of microorganisms that establish a biofilm on the material’s surface. The ability of microorganisms to form biofilms on almost any type of surface, whether natural or artificial, contributes to the considerable economic and environmental implications associated with MIC (Beech et al., 2014; Procópio, 2019; Pal and Lavanya, 2022; Javed et al., 2024). Indeed, the financial burden of corrosion-related damages and their associated costs is estimated to reach hundreds of billions of USD annually (NACE, 2024), of which MIC could account for as much as 20% (Heitz et al., 1996).

Microorganisms with different metabolic traits, such as manganese-oxidizing bacteria (MOB), acid-producing bacteria (APB), sulfur-oxidizing bacteria (SOB), nitrate-reducing bacteria (NRB) and acidophilic archaea, have all been shown to play a role in MIC, both in aerobic and anaerobic environments (Hubert et al., 2005; Rempel et al., 2006; Ashassi-Sorkhabi et al., 2012; Dong et al., 2018; Lahme et al., 2019; Qian et al., 2020). Nevertheless, sulphate-reducing bacteria (SRB), a ubiquitous group of Gram-negative bacteria capable of dissimilatory reduction of SO_4_^−^ to S^2−^, are often identified as the fingerprint in cases of corrosion failure due to MIC (Wu et al., 2019; Chen L. et al., 2021; Chen et al., 2022).

Understanding the mechanisms of MIC is extremely challenging due to the wide variety of processes coupled with the complexity of the microbial communities’ composition and environmental conditions. Consequently, accurate diagnoses, predictions and mitigations are still far from being achieved (Little et al., 2020; Wood et al., 2021; Dang et al., 2022; Wang et al., 2023). These limitations are further magnified when MIC occurs in extreme environments colonized by highly adapted microorganism, such as the deep sea.

The deep sea is typically defined as the body of water at depths greater than 200 meters, and is characterized by chemical and physical conditions distinct from those found in surface waters, such as lack of light, constant low temperatures, higher salinity and extremely high hydrostatic pressure (HHP) (Yayanos, 1986; Gage and Tyler, 1991; Fang et al., 2010), making the replication of the actual environmental conditions in laboratory-based experiments and, consequently, the achievement of a consensus on the mechanisms involved, extremely challenging (Wu et al., 2025).

As a result, corrosion in the deep sea has been rarely investigated, but with the increasing exploitation of the deep sea for industrial and scientific purposes, understanding the severity of this phenomenon has become essential (Chen S. et al., 2021; Murthy et al., 2023).

Previous research has mainly focused on the effect of HHP on corrosion in abiotic conditions (Zhang et al., 2016; Yang et al., 2017; Liu et al., 2022; Wu et al., 2025), with limited information about impact of MIC.

Nevertheless, biofilm formation on artificial materials, such as metallic plates or shipwrecks, in deep-sea environments has been extensively studied (Guezennec et al., 1998; Venkatesan et al., 2003; Bellou et al., 2012; Hamdan et al., 2021). Notably, Deltaproteobacteria are consistently associated with both metal and wood surfaces (Mugge et al., 2019; Moseley et al., 2022; Rajala et al., 2022).

Within this class, many SRB not only thrive in deep-sea environments, but also possess unique genes that are markedly different from those typically identified in other shallow-water SRB (Bale et al., 1997; Audiffrin et al., 2003; Khelaifia et al., 2011; Pradel et al., 2013; Guan et al., 2015; Zheng et al., 2021). This suggests that there might be a significant influence of HHP on sulfate reduction pathways, yet it remains unclear whether this can be reflected in the corrosion capabilities of these microorganisms.

This knowledge gap primarily stems from the high costs and practical challenges associated with field experiments, which often also suffer from a lack of abiotic controls and, to date, only a single study has conclusively attributed the corrosion rate and pits formation on a mooring chain deployed at a depth of 2,000 meters to MIC (Rajala et al., 2022).

The constraints and difficulties associated with field experiments underscore the importance of laboratory-based studies for gaining insights into the distinctive characteristics of MIC in deep-sea environments.

In this study, a novel high-pressure bio-electrochemostat was designed and assembled in order to overcome the limitations encountered in classic closed-batch experiments, such as the inability to provide a fresh supply of nutrients, to eliminate metabolic by-products, to remove dead cells from the biofilm and to control the partial pressure of dissolved gases. Recent findings on the severity of MIC under high-pressure conditions may, in fact, be influenced by experimental artifacts, as gas headspace is often utilized to create and maintain pressure during the culturing process (Li et al., 2025). While the advantages of employing continuous systems for cultivating microorganisms under HHP conditions have been previously established (Jannasch et al., 1996; Wirsen and Molyneaux, 1999; Houghton et al., 2007; Foustoukos and Pérez-Rodríguez, 2015), to the best of our knowledge, this device is the first specifically designed for studying MIC under HHP and continuous-flow conditions.

Using our device, the corrosive capabilities of *Pseudodesulfovibrio profundus* were evaluated under two different pressure conditions: atmospheric pressure (0.1 MPa) and high-hydrostatic pressure (30 MPa), which simulates the environment at a depth of 3,000 meters. The tests were conducted on marine-grade carbon steel.

The rate and severity of MIC were shown to be non-dependent on the biofilm’s cell density but, instead, on the metabolic state of the cells. Through both computational modeling and experimental findings, we show the crucial role of flow in influencing both biotic and abiotic corrosion rates.

Our results advocate for an improved experimental setup to evaluate MIC, which places more focus on preserving metabolic activity and minimizing corrosion product accumulation, thereby questioning the reliability of traditional methodologies in accurately gauging risk from MIC in the marine environment and the deep sea.

## 2 Methods

### 2.1 Microorganisms, testing media, and metal coupons

*Pseudodesulfovibrio profundus* strain 500–1 (DSMZ-German Collection of Microorganisms and Cell Cultures GmbH, Germany), a piezophilic sulfate-reducing bacterium (SRB) previously found in corrosive biofilms (Lanneluc et al., 2015) was employed as model microorganism. This SRB was first isolated from deep-sea sediments at a depth of 1,000 meters in the Japan Sea, and displays its highest metabolic activity at 15 MPa (Bale et al., 1997).

Cultures were grown anaerobically from glycerol stock in 10 mL *Desulfobacter* sp. Medium 195c (DSMZ-German Collection of Microorganisms and Cell Cultures GmbH, Germany) at room temperature and 0.1 MPa for 48 h. Successively, 40 mL of *Desulfobacter* sp. Medium 195c were added, and the cultures were incubated at room temperature and the designated experimental pressure (0.1 or 30 MPa), until exponential phase was reached.

Cells were harvested by centrifugation at 10000 x G for 8 min at 4°C and resuspended in anaerobic artificial sea water (ASW) medium 1 (National Center of Marine Algae and Microbiota, USA) (composition: 471 mM NaCl, 56.5 mM MgCl_2_, 27.5 mM MgSO_4_•7H_2_O, 9.7 mM KCl, 6.8 mM CaCl_2_•2H_2_O, 287 μM K_2_HPO_4,_ 10 mM NaHCO_3_ and 0.1% of Mineral and Wolfe’s vitamins solution) to a final OD_600_ of 0.02.

ASW was sterilized by autoclaving at 121°C for 12 min. Successively, 0.22 μm filter sterilized 2.2 mM L-lactate, 40 mM 3-(N-Morpholino) propanesulfonic acid sodium salt (MOPS) and 100 ppm L-cysteine were added as organic carbon source, pH buffer and oxygen scavenger, respectively.

Finally, the solution was deoxygenated by bubbling with filter-sterilized 95% N_2_ + 5% CO_2_ 45 min l^−1^ and the pH was adjusted to 7.8 through the addition of NaOH. The measure of the DO was obtained with a DO meter (Mettler Toledo, USA) with a maximum accepted limit of 0.05 ppm.

The discrepancy between the final salinity of the medium and the average concentration in deep-sea environments (~598 mM) is unlikely to introduce significant bias, given the minimal influence of salinity on the corrosion process (Chohan et al., 2024).

Growth and sulfate consumption analyses were conducted in batch cultures using the same ASW at both experimental pressures without the addition of the metal coupons. These served as a baseline to assess the impact of hydrostatic pressure on the growth and metabolism of the model organism.

Corrosion tests were performed on 10 × 10 × 2 mm marine grade AH36 mild steel coupons (composition shown in Supplementary Table S1), polished sequentially to US grit 280, 360 and 800, washed with acetone and UV sterilized for 30 min/face before incubation.

Working electrodes (WE) for electrochemical analysis were prepared covering one face and all sides of a polished coupon in epoxydic resin resulting in a final exposed area of 1 cm^2^.

### 2.2 High pressure bio-electrochemostat

**Figure 1:**
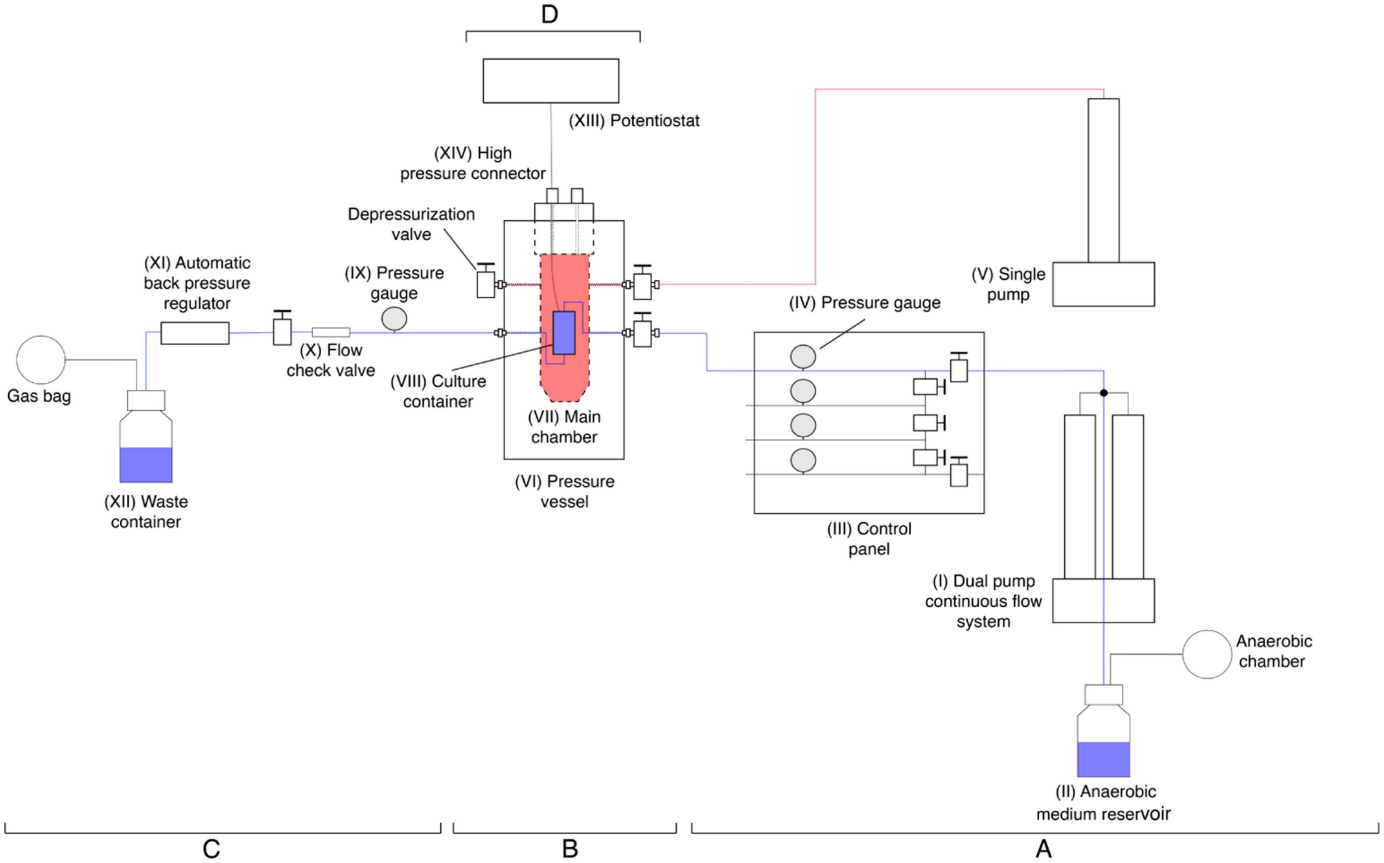
Schematic drawing of the high-hydrostatic pressure bio-electrochemostat. In blue is represented the line of the medium from the pump (I) to the waste collection (XII), in red is highlighted the pressure vessel main chamber pressurization line. The pre-reactor **(A)** is constituted by two high-pressure pumps (I, V), a fresh medium reservoir (II), and the control panel with pressure gauges (III, IV). The reactor **(B)** includes the pressure vessels (VI) with its main chamber (VII) and the POM culture container inner chamber (VIII). The post-reactor **(C)** consists of a pressure gauge (IX), flow check valve (X), ABPR (XI) and waste container (XII). The potentiostat **(D)** includes the instrument (XIII) and the high-pressure connector (XIV).

A novel bio-electrochemostat was designed and manufactured to grow microbial cultures in HHP conditions in a continuous-flow system set up (Figure 1; Supplementary Figure S1).

The device is composed of four main components:

- Pre-reactor (A).
- Reactor (B).
- Post-reactor (C).
- Potentiostat (D).

#### 2.2.1 Pre-reactor

The pre-reactor consists of two separated pumps. A 65D Hastelloy C-276 dual-pump continuous-flow system (Teledyne ISCO, USA) (I), which uses a 2 liters glass bottle capped with gas proof sealing as fresh medium reservoir (II) and supplies the fresh medium at constant flow rate while maintaining the set hydrostatic pressure. Followed by a control panel (III) that allows for the redirection of the medium in four separate lines, each one equipped with a pressure gauge (IV) allowing to run parallel experiments.

The second pump consists in a single Nitronic 50 stainless steel pump (Teledyne ISCO, USA) (V) which pressurizes the main chamber of the reactor (VII).

#### 2.2.2 Reactor

The reactor is a custom-designed pressure vessel made of 304 stainless steel (HiP High Pressure Equipment, USA) (VI). Inside the main chamber (VII), there is a secondary container made of polyoxymethylene (POM) (VIII), equipped with a rubber cap to facilitate electrochemical connections. This inner container is connected to the dual pump and houses the bacterial culture along with the corrosion specimens.

#### 2.2.3 Post reactor

The post reactor includes a pressure gauge (IX) which ensures the homogeneity of the pressure before and after the culture, followed by a flow check valve (X) to avoid any back flow of used medium.

The system is completed with an ABPR10 automatic back pressure regulator (DCI Test System, USA) (XI), which maintains the pressure of the continuous flow line constant. A container for the waste medium is placed at the end of the line (XII).

#### 2.2.4 Potentiostat

A potentiostat (XIII) is directly connected to the incubator through a high-pressure connector (XIV) fixed to the top of the pressure vessel.

In order to confirm the ability of the device to maintain an anaerobic and, when required, sterile environment within the entire system, dissolved oxygen (DO) was measured at the conclusion of each incubation period in the leftover medium reservoir as well as in the culture and waste medium, and sterility was confirmed through DNA extraction from the medium and the coupons surface.

### 2.3 Experimental setup

Each experiment was conducted using either *P. profundus* cultures or sterile medium at room temperature (typically about 24°C). The decision to use a temperature different from the average deep-sea temperature was necessary to obtain significant results within the constrained timeframe of laboratory experiments. The experiments duration was 10 days, and they were conducted under four distinct conditions: static or continuous flow (0.5 mL min^−1^) and 0.1 or 30 MPa (Supplementary Table S2). All experiments were conducted using the HHP bio-electrochemostat.

The *P. profundus* cultures or the sterile medium were added in the inner POM containers (Figure 1) under anaerobic conditions and subsequently connected to the system previously disinfected with 10% (by weight) sodium hypochlorite, rinsed with sterile MilliQ water and flushed with anaerobic sterile medium.

For each condition, four AH36 steel coupons were employed, and two sets of independent biological replicates were conducted to ensure robust data collection and analysis.

A 24-h static period was implemented before introducing continuous flow to promote bacterial attachment and biofilm formation on the steel coupons, considering the short incubation duration. During the following nine days a constant flow was applied at a rate of 0.05 mL min^−1^, corresponding to a final hydraulic retention time (HRT) of 25 h.

The choice of the flow rate was tailored to suit the growth rate of the model bacteria while minimizing the increase in abiotic corrosion caused by medium perturbation.

Out of the four coupons, two coupons were dedicated to the mass loss analysis and corrosion characterization, one was used for FESEM and EDS and one for live/dead bacterial staining.

The same incubation setup was utilized for electrochemical analysis, with the incubation period shortened to 7 days, as the growth and metabolic activity in static conditions were showed to reach a plateau after 72–96 h (Supplementary Figure S2).

### 2.4 Weight loss analysis and corrosion characterization

Corrosion rate was calculated following ASTM G1-03 protocol (ASTM, 2107). Each coupon was weighed in triplicates before the experiment using a XP2U microbalance (Mettler Toledo, USA). After the incubation, coupons were sonicated for 10 min in 1% HCl + 0.35% hexamethylene tetramine to remove corrosion product. The weight was recorded again, and the calculated mass loss applied to the Equation 1:


(1) $$Corrosionrate=\frac{\left(K\times W\right)}{\left(A\times T\times D\right)}$$

where K, constant (0.876 × 10^4^), W, mass loss (g), T, time of exposure (hours), A, area (cm^2^) of coupon, D, density of the steel (g cm^−3^).

Statistical significance of differences in corrosion rates was assessed using pairwise *t*-tests with Bonferroni correction.

Topographical analyses of the coupon surfaces were conducted using a VK-X1100 3D laser scanning confocal microscope (CLSM) (Keyence, Japan) to identify the corrosion features specific to each condition.

### 2.5 Biofilm and corrosion product analyses

LIVE/DEAD BacLight Bacterial Viability Kit (Thermo Fisher Scientific, USA) was used to stain the biofilm on the surface of the specimens after the immersion test following the manufacturer’s protocol. Successively, an AxioObserver 7 CLSM (Zeiss, Germany) was employed to investigate the bacterial distribution and to differentiate live from dead cells, which were counted within randomly selected areas and scaled up to the surface of one face of the specimens (1 cm^2^).

A JSM-7800F FESEM (JEOL, Japan) coupled with an Xmax 150 mm^2^ EDS detector (Oxford Instruments, UK) was employed to obtain images and elemental composition of the biofilm/corrosion product after samples fixation in 2.5% glutaraldehyde and dehydration in ethanol at increasing concentration (30, 50, 70, 80, 96, 100%) as described by Rajala et al. (2017).

### 2.6 Electrochemical analyses

Electrochemical measurements were conducted employing a VMP3 potentiostat (BioLogic, France) in a conventional three-electrode setup composed by an AH 36 steel coupon as the working electrode (WE), a silver wire as the pseudo reference electrode (RE), and a platinum coiled wire as the counter electrode (CE). The coupon was attached to a current collector (copper wire) through conductive copper tape and then covered by insulating epoxy resin to avoid any signal from the current collector.

LPR and EIS measurements were used to monitor the corrosion processes following the same experimental setup and under all conditions applied on the previous tests (Supplementary Table S2). Each replicate consisted in a coupon immersed in the sterile medium or bacterial culture together with RE and CE for a period of 7 days.

LPR measurements were recorded every 24 h while EIS data were collected every 6 h.

LPR is commonly used to measure corrosion rate. While the calculation of corrosion current requires additional information on the reaction rates (e.g., Tafel constants), which are not easy to determine in MIC contexts, the polarization resistance (*R*_p_) can be determined simply as the slope of the I/E curve in the proximity of the E_corr_ and used as indicator of the corrosion current. Higher *R*_p_ corresponds to lower I_corr_ and vice versa.

Analyses was assessed at ±20 mV relative to OCP with a scan rate of 0.125 mV s^−1^. For electrochemical impedance spectroscopy (EIS) measurements, an alternating current (AC) signal with an amplitude of 10 mV vs. OCP and a frequency range spanning from 1 MHz to 10 mHz was applied.

The data exhibited pronounced noise, especially at higher frequencies, resulting in some datasets being uninterpretable. However, the EIS data at 30 MPa were of sufficient quality to allow equivalent circuit fitting using the software EC-Lab (BioLogic, France). These results were modeled using a one time-constant equivalent circuit (see Supplementary Figure S3), which provided a good representation of most of the spectrum from 24 to 168 h (Supplementary Figure S4). In this model, Q2 represents the constant phase element (CPE), commonly used to describe non-ideal capacitive interfaces, such as those typically encountered in MIC systems.

The effective capacitance C*_eff_* was calculated from the parameters of the constant phase element (CPE) using the Brug’s equation (Equation 2) (Dominguez-Benetton et al., 2012):


(2) $$C_{eff}=Q^{1/\alpha }{\left(\frac{R_{e}R_{t}}{R_{e}+R_{t}}\right)}^{\left(1-\alpha \right)/\alpha }$$

where Q is the capacitance, α is a dimensionless parameter associated with the distributed behaviour of the process, $R_{e}$ is the resistance of the electrolyte and $R_{t}$ is the charge-transfer resistance at the electrode/electrolyte interface.

### 2.7 Dimensional analysis on the corrosion rate

The impacts of the flow and SRB activity on the corrosion rate were assessed considering the corrosion process as a mass transfer problem in which the corrosion products are transported from the surface of the coupons to the surrounding fluid. The corrosion rate is positively correlated to the concentration gradient of the product around the coupon.

The analysis first estimated the diffusion coefficient of the substance, *D* in the unit of m^2^ s^−1^, without any background flow (Supplementary Figure S5A). According to the Fick’s second law (Fick, 1855), the concentration of the corrosion products, 𝜑 in the unit of mol m^−3^, is governed by


(3) $$\frac{\partial \varphi }{\partial t}=D\nabla^{2}\varphi$$

where *t* is time and *𝛻* is a vector differential operator in the three-dimensional domain. The characteristic concentration, length and time were defined to be *Φ*, *L* and *T*, respectively, and hence the 𝜕𝜑/𝜕*t* and 𝛻^2^𝜑 terms in Equation 3 were scaled as *Φ*/*T* and *Φ*/*L*^2^, respectively. Therefore, the diffusion coefficient *D* could be scaled as *LV_d_*, where *V_d_* = *L*/*T* was the typical diffusion speed of the corrosion products.

Once the diffusion coefficient is derived from the experimental measurements, the dimensional analysis is then performed to address the effect of continuous flow on the corrosion rate of the coupon. In addition to diffusion, the background flow removed the corrosion products formed on the coupon’s surface, which lead to a larger concentration gradient of corrosion products and a faster corrosion rate.

The corrosion rate in the continuous flow condition, scaled as *L*/*T*, was solved by the applying the Newton’s law of cooling for mass transfer (Newton, 1701), which expresses the mass transfer rate of the corrosion products from the coupon surface to the bulk fluid by


(4) V∂φ∂t=hmA(φs−φ∞)

where V and A were the volume and the surface area of the coupon, respectively, h_m_ was the convective mass transfer rate in the unit of m s^−1^, and $\left(\varphi_{s}-\varphi_{\infty }\right)$ was the difference in concentration between the surface and bulk fluid which could be scaled as *Φ* as well. Another dimensional analysis performed on Equation 4 by using the same characteristic concentration, length and time concluded that the corrosion rate in the continuous flow condition could be scaled as *h_m_*.

Lastly, *h_m_* was derived by modeling the experimental setup as a convective mass transfer problem due to the two-dimensional flow over the coupon (Supplementary Figure S5B). The latter is modeled as a flat plate. For ASW flowing past the flat plate of length *l* = 10 mm at the volume flow rate *Q* = 0.05 mL min^−1^, the Reynolds number (which defines the ratio of the inertia force to the viscous force exerted on the fluid) was found to be Re = *Vl*/*ν* = 5.3 ⨯ 10^−3^, where *ν =* 1.64 ⨯ 10^−6^ m^2^ s^−1^ is the kinematic viscosity of ASW at 4°C (ITCC, 2011) and *V* = *Q*/(*πd*^2^/4) was the averaged flow velocity in the main chamber of diameter *d* = 35 mm (VII in Figure 1). Therefore, the flow past the coupon was well within the laminar flow regime. The equations that govern the steady-state fluid motion within the velocity boundary layers are the continuity equation (Equation 5) and *x*-momentum equation (Equation 6) for incompressible fluid:


(5) $$\frac{\partial u_{x}}{\partial x}+\frac{\partial u_{y}}{\partial y}=0$$

(6) $$u_{x}\frac{\partial u_{x}}{\partial x}+u_{y}\frac{\partial u_{x}}{\partial y}=\nu \frac{\partial^{2}u_{x}}{\partial y^{2}}$$

where *u_x_* and *u_y_* are the flow velocities in the *x* and *y* directions, respectively. The convective mass transfer of the substance is governed by the species conservation equation:


(7) $$u_{x}\frac{\partial \varphi }{\partial x}+u_{y}\frac{\partial \varphi }{\partial y}=D\frac{\partial^{2}\varphi }{\partial y^{2}}$$

For a flat plate in parallel flow, if the Schmidt number (which defines the rate of the viscous momentum transfer relative to the molecular diffusion of the substance) Sc = *ν*/*D* is much larger than 1, the averaged Sherwood number (which represents the ratio of the convective mass transfer rate to the diffusion rate) could be derived as Sh = *h_m_l*/*D* (Incropera et al., 1996). This Sh could further be expressed as 0.664 Re^1/2^Sc^1/3^ (Incropera et al., 1996). Therefore, the convective mass transfer rate *h*_m_ was given by:


(8) hm=0.664DRe1/2Sc1/3l=0.664V1/2Sc1/3l1/2ν1/6

Since corrosion rate in the continuous flow condition was scaled as *h_m_* from the dimensional analysis on Equation 4, one could use Equation 8 to estimate the corrosion rate in the presence of flow under the condition that Sc ≫ 1.

## 3 Results

### 3.1 Corrosion rates

**Figure 2:**
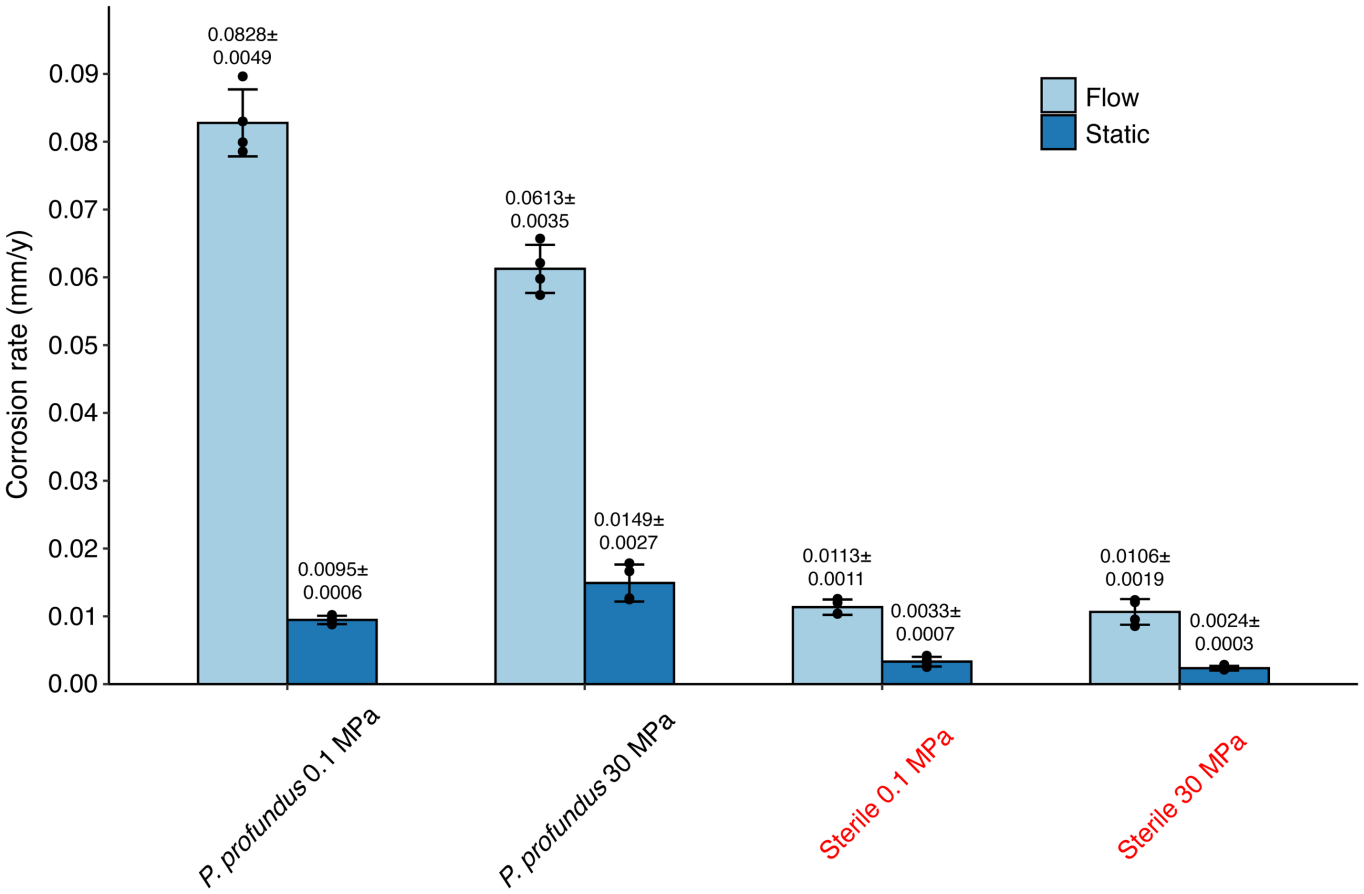
Corrosion rates calculated through weight loss measurements in all conditions after 10 days of incubation and corrosion product removal. The error bars indicate the standard deviation calculated on the 4 replicates.

The quantification of corrosion rates after a 10-day period, encompassing all eight tested conditions, was conducted using weight loss analysis (Figure 2).

The presence of *P. profundus* typically led to a rise in corrosion rates compared to the sterile conditions, and the corrosion was further exacerbated in the presence of continuous flow. The highest recorded corrosion rate was measured in the *P. profundus* continuous-flow culture at 0.1 MPa (0.083 ± 0.005 mm y^−1^) while the lowest was associated with the static sterile medium at 30 MPa (0.002 ± 0.0003 mm y^−1^).

HHP did not alter the corrosion in the sterile medium (*p* = 0.1094 under static conditions and *p* = 0.2227 under continuous flow). However, it significantly increased (*p* < 0.05) the severity of MIC under static conditions (0.015 ± 0.003 mm y^−1^) while reducing it in continuous-flow conditions (0.061 ± 0.003 mm y^−1^).

### 3.2 Characterization of corrosion product morphology and elemental composition

**Figure 3:**
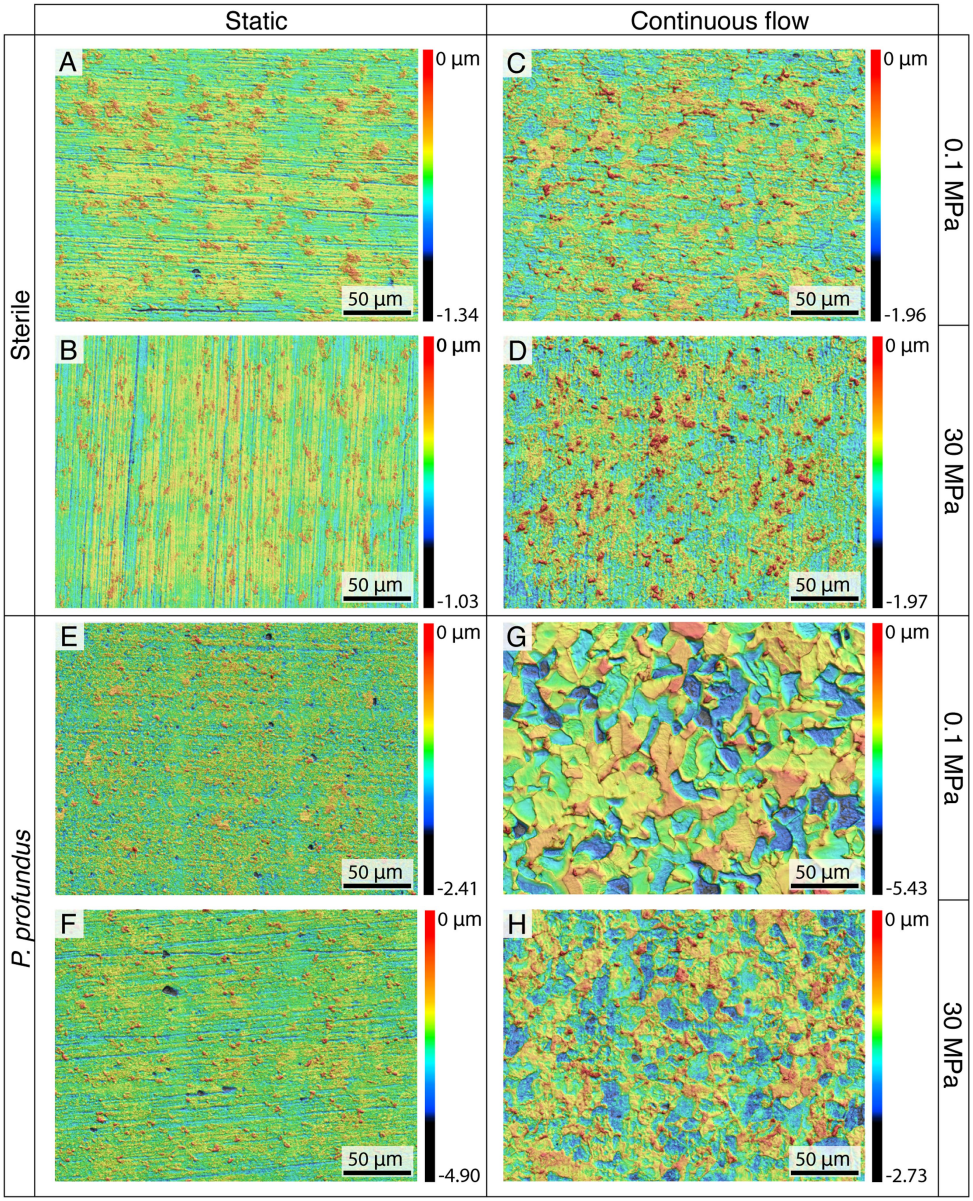
Topographical analyses of the surface of the coupons incubated at 0.1 **(A,C,E,G)** and 30 MPa **(B,D,F,H)** after corrosion product removal using CLSM.

To examine the topography of the surface of the coupons after the removal of biofilm and corrosion products, 3D CLSM was employed (Figure 3).

In sterile conditions, no discernible differences related to hydrostatic pressure were observed (Figures 3A–D).

Nevertheless, after static incubation, the characteristic traces of the polishing process were still clearly visible (Figures 3A,B), whereas incubation in continuous-flow conditions caused the coupon surfaces to exhibit an increased corrosion level characterized by the complete removal of the superficial layer (Figures 3C,D).

The corrosion in *P. profundus* cultures was characterized by the formation of small pits in static conditions (Figures 3E,F), with similar maximum pit depths between atmospheric pressure and HHP (2.81 and 2.02 μm respectively) but double maximum widths in those produced at HHP (6.08 and 12.26 μm respectively) (Supplementary Figure S6).

On the other hand, under continuous-flow conditions, the surfaces of the specimens exhibited the typical features of generalized corrosion, showing a uniform distribution without any significant pit (Figures 3G,H).

The corrosion products in presence of the sterile medium was not visible under naked eyes (Supplementary Figure S7) and its elemental composition remained consistent across all conditions, with more than 60% (wt%) represented by iron (Fe) and the remaining 40% containing oxygen (O), phosphate (P) and other elements (Table 1).

**Table 1:** The elemental composition as wt% of the corrosion product was analyzed through EDS.

	Sterile				
	Fe	O	P	S	Other
Static 0.1 MPa	64.4 ± 3.0	28.3 ± 1.8	1.6 ± 0.5	N/D	5.7 ± 5.3
Static 30 MPa	64.4 ± 1.3	28.4 ± 0.6	1.0 ± 0.2	N/D	6.2 ± 2.1
Flow 0.1 MPa	63.3 ± 3.8	26.8 ± 5.3	2.6 ± 1.2	N/D	4.8 ± 7.8
Flow 30 MPa	61.6 ± 1.9	31.1 ± 1.2	1.0 ± 0.6	N/D	6.3 ± 3.7

	*P. profundus*				
	Fe	O	P	S	Other
Static 0.1 MPa	49.7 ± 0.5	19.7 ± 0.7	3.3 ± 0.2	20.0 ± 0.4	7.3 ± 1.8
Static 30 MPa	46.6 ± 1.9	24.4 ± 1.8	3.6 ± 0.9	14.3 ± 1.2	11.1 ± 5.8
Flow 0.1 MPa	62.9 ± 2.0	13.6 ± 1.3	3.2 ± 0.5	12.9 ± 0.3	7.4 ± 4.1
Flow 30 MPa	55.0 ± 0.8	26.3 ± 0.9	1.5 ± 1.3	13.9 ± 1.8	3.3 ± 4.8

The error represents the calculated standard deviation out of 4 measurements from each of the 2 biological replicates. N/D, not detectable.

However, microbial activity consistently produced a black-colored corrosion product, which exhibited visibly characteristic roughness under flow or static conditions (Supplementary Figure S7). The elemental composition exhibited a more variable percentage of Fe (between 46.6 ± 1.9 and 62.9 ± 2.0) and a concentration of O (between 13.6 ± 1.3 and 26.3 ± 0.9) which seemed to be consistently higher at HHP, with a significant amount of sulfur (S) always present (between 12.9 ± 0.3 and 20.0 ± 0.4) (Table 1).

Notably, S concentration was found to be higher in static conditions and, more specifically, at atmospheric pressure (20.0 ± 0.4) where it was more than 25% higher than at 30 MPa.

### 3.3 Evaluation of biofilm viability and density

**Figure 4:**
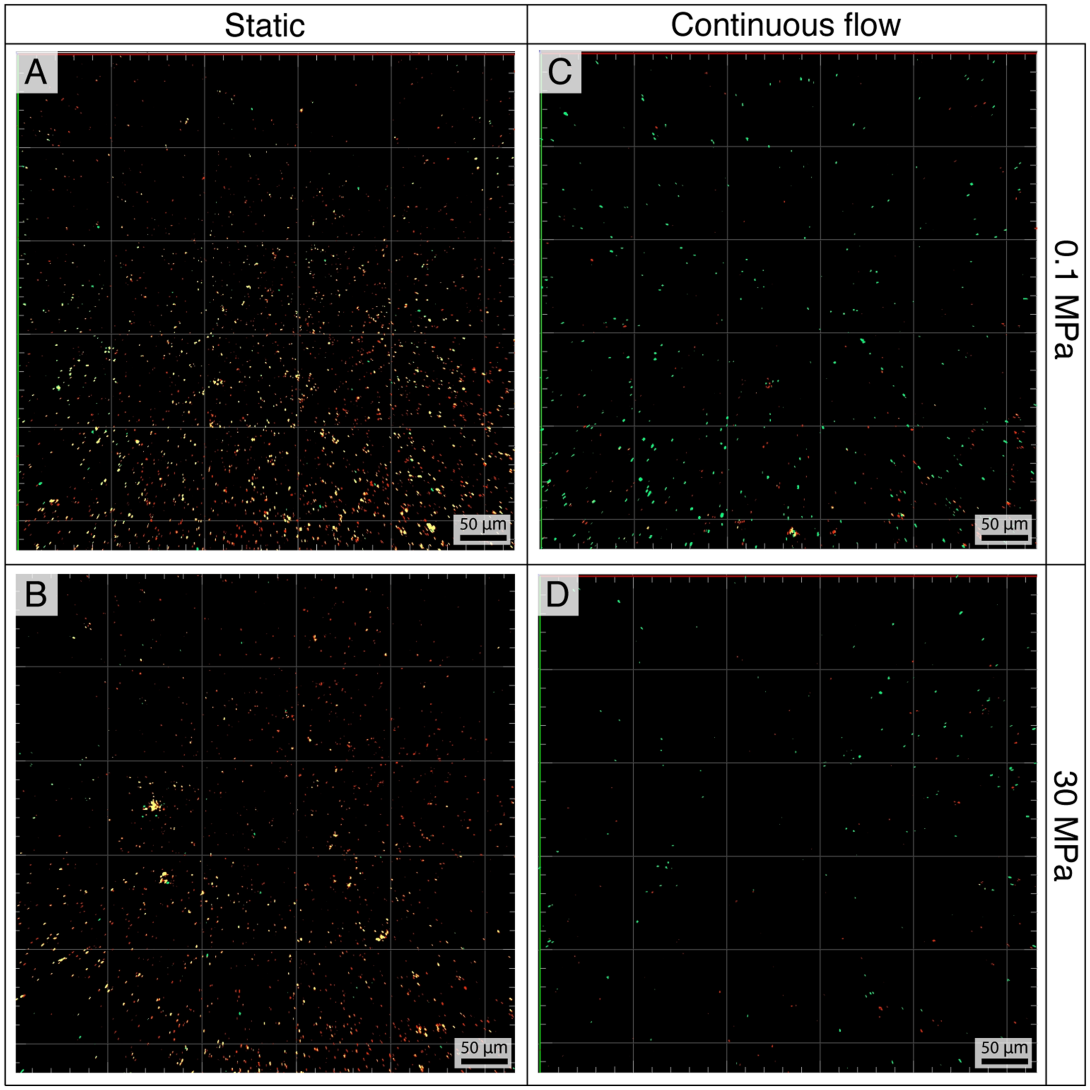
*Pseudodesulfovibrio profundus* biofilms in static **(A,B)** and flow **(C,D)** conditions at 0.1 MPa **(A,C)** and 30 MPa **(B,D)** stained using Syto9 and Propidium Iodide. With this technique live cells appear in green while dead or damaged cells appear in red or yellow.

Figure 4 shows the fluorescence CLSM images of the biofilm formed on coupons after 10 days of incubation. The largest concentration of cells was observed in static conditions (Figures 4A,B), while increased hydrostatic pressure corresponded to a lower abundance of bacteria (Figures 4B–D). Supplementary Table S3 summarizes the average abundances in cells mm^−2^.

The viability of the cells was evaluated using Syto9 and Propidium Iodide staining to differentiate between live and dead/damaged cells, represented in green and red/yellow, respectively.

The ratio of live versus dead cells in flow conditions was 2.42 ± 0.72 at 0.1 MPa and 1.92 ± 0.54 at 30 MPa. On the contrary, the ratio was opposite in static conditions, with a higher amount of dead or damaged cells over the living ones (live vs. dead, 0.06 ± 0.08 and 0.05 ± 0.05 at 0.1 and 30 MPa respectively).

CLSM was also used to visualize the biofilm matrix, which in flow conditions was constantly more abundant regardless of the pressure (Supplementary Figure S8).

**Figure 5:**
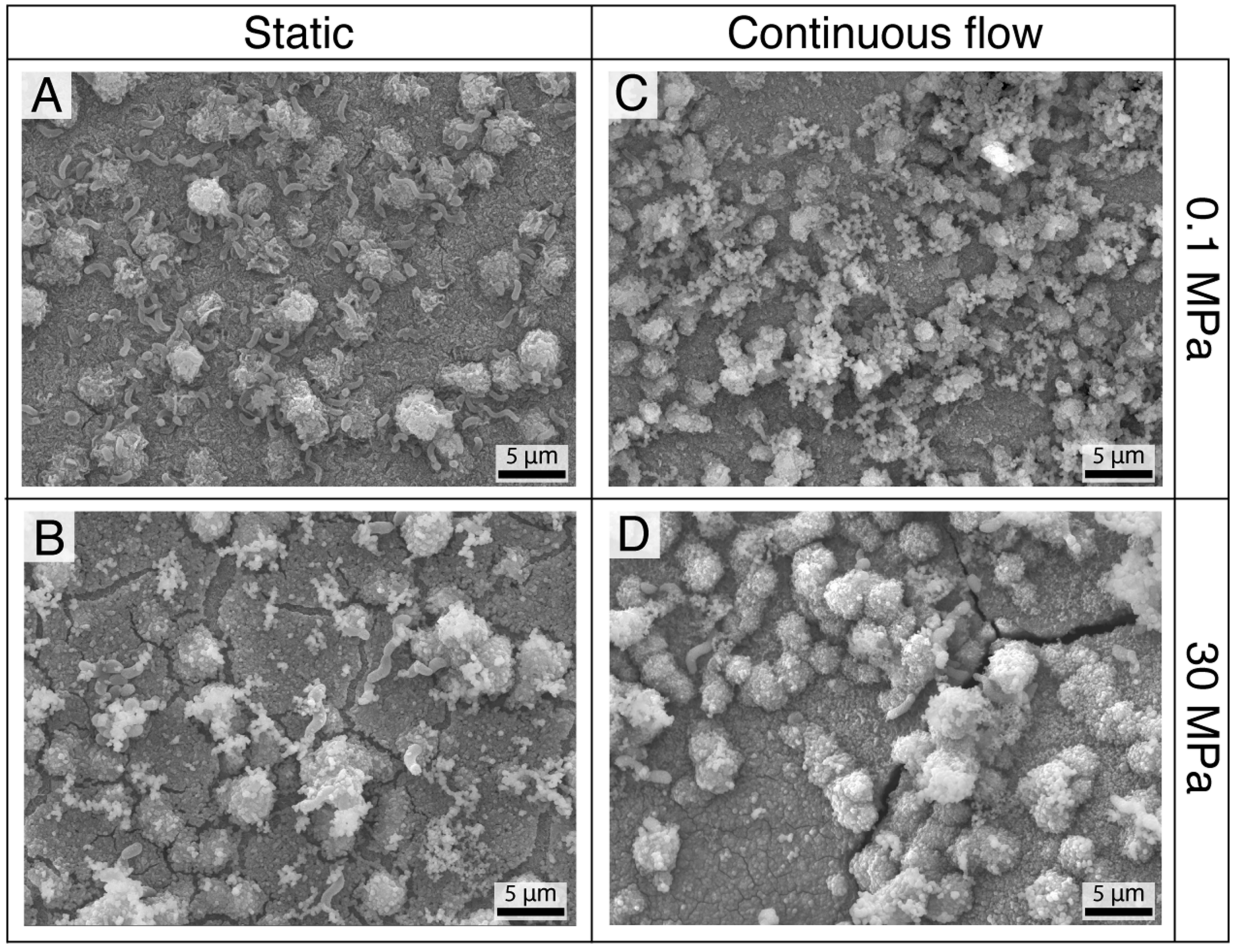
FESEM analysis of the biofilm after 10 days of incubation in static **(A,B)** and flow **(C,D)** conditions at 0.1 **(A,C)** and 30 MPa **(B,D)**.

The biofilm was further investigated under field emission scanning electron microscope (FESEM) after dehydration (Figure 5). The images confirmed the results of the CLSM with higher abundance of sessile cells in static conditions (Figures 5A,B) contrasted by higher matrix production in presence of flow (Figures 5C,D). Interestingly, at HHP the cells were notably larger in size compared to those as atmospheric pressure (Figures 5B–D).

Furthermore, the corrosion products of samples incubated at 30 MPa were characterized by the presence of distinctive features and cracks (Figures 5B–D), some of which could also be observed on the corrosion product under sterile conditions.

### 3.4 Electrochemical analyses

**Figure 6:**
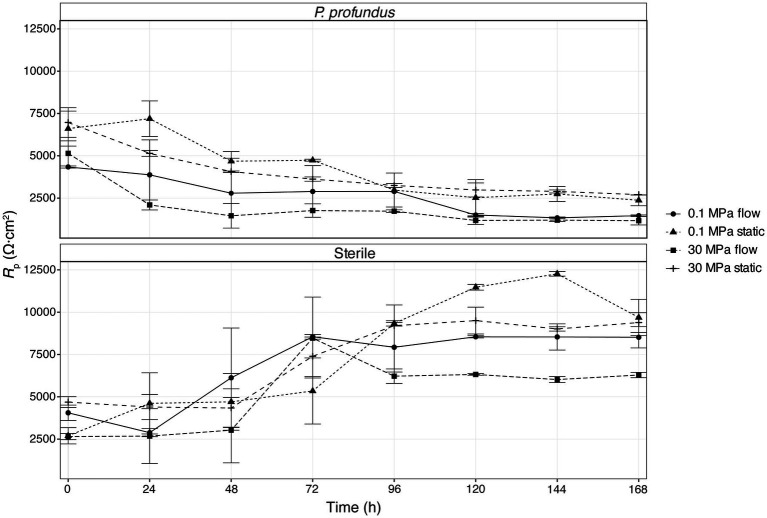
LPR determined from linear polarization during a 7-day incubation period for all conditions tested. The error bars represent the standard error calculated on the two biological replicates.

Linear polarization resistance (LPR) measurements were conducted under both biotic and abiotic conditions at high and low pressure. While similar trends as a function of incubation time were observed in both the sterile medium and *P. profundus* cultures, the most pronounced differences within each group were between flow and non-flow conditions (Figure 6; Supplementary Table S4). The highest *R*_p_ values were observed in samples incubated under sterile medium and static conditions, whereas the lowest values were recorded for coupons immersed in *P. profundus* cultures under flow conditions. Notably, the effect of flow on *R*_p_ was much higher than that of pressure, especially under biotic conditions.

In sterile conditions, the *R*_p_ increased consistently across all samples during the initial 48/72 h under continuous flow, and until 96/120 h under static conditions. In samples inoculated with *P. profundus,* the *R_p_* decreases sharply within the first 48 h in all replicates, and particularly under flow conditions, consistently with the higher corrosion rate observed in biological experiments (Figure 6; Supplementary Table S4). The effect of pressure is negligible, similarly to what was observed for the corrosion rate. The R_p_ decreases steadily at longer times for all the conditions applied, although much slower under sterile conditions, indicating that the chosen experimental time is sufficient to capture the evolution of the MIC process.

**Figure 7:**
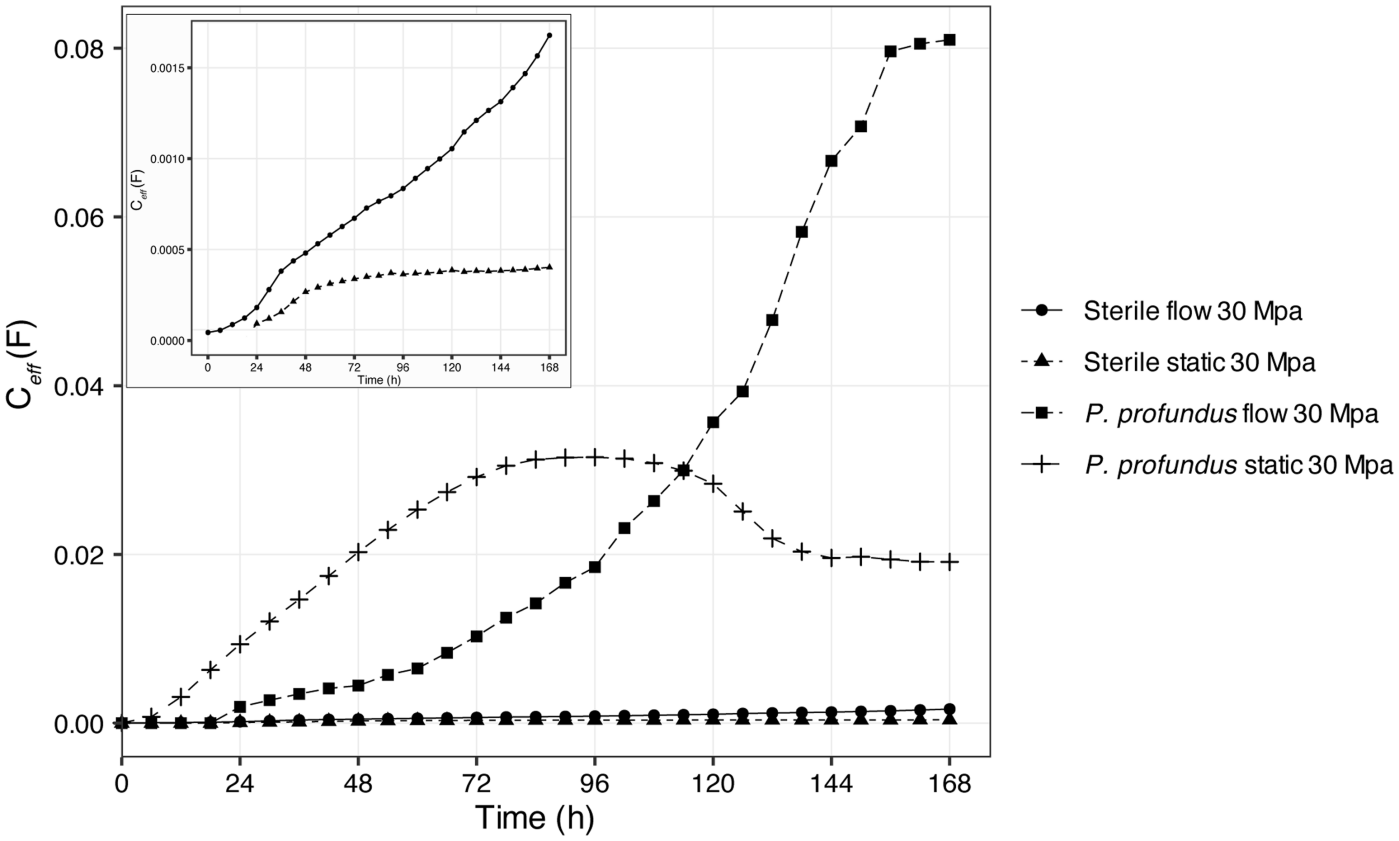
C*_eff_* calculated using the electrochemical impedance spectroscopy (EIS) equivalent circuit under high-pressure conditions along a 7-day incubation period (representative results were selected). The box illustrates a detailed view of the two sterile samples.

Under sterile conditions, C*_eff_* remained consistently low, with a notable and steady rise in presence of flow. In contrast, it showed only a slight increase before stabilizing after approximately 72 h under static conditions (Figure 7). However, in presence of *P. profundus*, C*_eff_* exhibited much higher values following similar trends. Under flow conditions, C*_eff_* continued to increase throughout the entire duration of the experiment, while, in static conditions, a slight decline and eventual plateau followed an initial increase (Figure 7).

### 3.5 Theoretical model

The theoretical model based on the dimensional analysis was adopted to estimate the corrosion rate in continuous flow condition. Treating the corrosion process as a mass transfer question, the corrosion products are transported from the surface of the coupons to the surrounding fluid. The concentrations of the corrosion products are highest at the surface of the plate, and approach to zero on the channel wall. The typical length scale for a mass transfer problem may be defined as the volume–surface ratio of a body, which is the thickness of the flat plate (Coulson et al., 1990; Kunes, 2012) in our analyses, *L* = 2 mm.

The typical diffusion speeds of the corrosion products, *V_d_*, were determined to be the corrosion rates in static media, which were found to be around 0.002 mm y^−1^ and 0.010 mmy^−1^, respectively. Therefore, the scales of the diffusion coefficients of the corrosion products, D, which were scaled as *LV_d_* from Equation 2, were determined to be 1.3 ⨯ 10^−16^ m^2^ s^−1^ and 6.3 ⨯ 10^−16^ m^2^ s^−1^ for the sterile and biotic incubations, respectively.

It was found that, for both sterile and biotic cases, the Reynolds number of the flow in the main chamber, Re, was much smaller than unity while the Schmidt number, Sc = *ν*/*D*, where *ν* is the kinematic viscosity of the fluid, were found to be much larger than unity. Consequently, Equation 8 could be applied to estimate the corrosion rate in the presence of the flow. For the sterile incubation, substituting *D* = 1.3 ⨯ 10^−16^ m^2^ s^−1^, Re = 5.3 ⨯ 10^−3^, Sc = 1.3 ⨯ 10^10^ and coupon length *l* = 10 mm into Equation 8 the derived corrosion rate was 0.047 mm y^−1^.

For the biotic condition, in comparison, the corrosion rate was calculated to be 0.132 mm y^−1^ by using *D* = 6.3 ⨯ 10^−16^ m^2^ s^−1^, Re = 5.3 ⨯ 10^−3^, Sc = 2.2 ⨯ 10^9^ and *l* = 10 mm.

## 4 Discussion

This study demonstrated that, despite certain limitations—such as non-fully realistic temperature and salinity—the use of the high-pressure bio-electrochemostat under constant flow conditions provides a more accurate simulation of MIC in both shallow and deep-sea simulated conditions.

The capabilities of the novel instrument developed here highlight the inaccuracy of traditional sealed batch experiments, which, overlooking the increase in partial pressure of gases, the accumulation of corrosion residues, the consumption of nutrients and the accumulation of metabolic by-products, fail to fully capture microbial metabolic responses to environmental disturbances, often resulting in incomplete or misleading conclusions.

The impact of flow on biofilm was quantifiable with the CLMS analyses. The shear stress fostered uniformity in the biofilm, resulting in heightened thickness and density of EPS, and reduced biomass content as result of the dispersion of dead cells, as previously described (Brading et al., 1995; Krsmanovic et al., 2021).

The results from the continuous-flow system showed dramatic differences in corrosion rates, corrosion morphology, and bacterial activity when compared to conventional static setups. These findings emphasizes the importance of using dynamic, continuous-flow systems to more accurately simulate the environmental factors influencing MIC and to enhance our understanding of its real-world impact.

A notable increase in the corrosion rate under flow conditions was observed in both sterile and biotic conditions (Figure 2), a finding that aligns with earlier studies comparing static versus semi-continuous flow incubations (Taheri et al., 2005).

In the sterile experiments, HHP appeared to have a minimal, if not negligible, effect.

To date, reaching a consensus on its precise impact on the thermodynamics of corrosion processes remains challenging (Ma et al., 2022), since HHP affects the physicochemical properties of both the electrolyte and the material, as well as the solubility of gases.

Given that the corrosion reactions in the presence of bacteria do not fundamentally differ from those in abiotic environments, and that HHP had no noticeable impact on the metabolism and growth of *P. profundus* (Supplementary Figure S2), we hypothesize that, in our set up, the impact of HHP on microbial corrosion was primarily limited to its influence on bacterial attachment (Figures 4, 5).

The corrosion rates in the presence of SRB were approximately 5-fold higher than those observed in sterile medium (Figure 2), a rate also consistent with previous studies (Guan et al., 2021; Yang et al., 2022), but notably lower than rates reported in other works conducted with single SRB species grown in enriched media specific for this group of bacteria (Xu and Gu, 2014; Dou et al., 2019; Wang et al., 2021).

Nevertheless, in the interest of obtaining realistic conditions, the ASW used in this study was intentionally low in nutrients and organic carbon to mimic the oligotrophic nature of the deep-sea environment (Wirsen and Molyneaux, 1999; Parsekar and Jobby, 2023). Under these conditions, the growth of *P. profundus* was limited to only a few division cycles within the first 72 h followed by a stationary phase. At the same time, the dissimilatory reduction of sulfate reached a plateau after about 120 h of incubation (Supplementary Figure S2).

Some SRB can utilize the H_2_ evolved during the dissolution of iron as an electron source for sulfate reduction. This process leads to a continuous consumption of electrons, which creates a thermodynamically favorable microenvironment on the metal surface, promoting enhanced dissolution. This phenomenon is commonly referred to as electrical microbially influenced corrosion (EMIC) (Enning et al., 2012).

If EMIC was indeed the main mechanism involved in the corrosion process, under our experimental setup, characterized by a limited availability of organic carbon as electron donor, the corrosion rate would have increased under static conditions (Xu and Gu, 2014; Dou et al., 2019).

Conversely, the substantial rise observed under continuous-flow conditions, where new nutrients and organic carbon were constantly supplied, suggests that the dominant corrosion mechanisms involved in our study was of chemical nature (CMIC) and caused by the end-products of the bacterial metabolism. In the case of *P. profundus*, H_2_S, the final product of sulfate reduction, has been previously shown to act as catalyst for the corrosion process (Videla et al., 2005; Lv et al., 2021).

The corrosion rates estimated by the theoretical model appeared to be in the same order of magnitude but around 5 times higher than the experimental data (around 0.01 mm y^−1^). This was attributed to the fact that the model simplified the experimental setup to a classical mass transfer problem without considering the complex flow field over the coupon and detailed corrosion mechanism. Notably, the pressure variation did not contribute to the corrosion rate in our model, which is in line with the experimental finding that pressure has negligible effect on corrosion in the absence of SRB (Figure 2).

In the presence of *P. profundus*, the theoretical result only overestimated the corrosion rate by around 2-fold. This finding supports the hypothesis that, other than a mass transfer effect, the presence of a continuous flow was effective in replenishing available nutrients and organic carbon to the SRB biofilm and in removing the metabolic by-products, which ultimately resulted in a higher live-to-death cell ratio, lower cell density and a higher EPS production (Figures 4, 5; Supplementary Figure S8; Supplementary Table S3). As a consequence of the increased corrosion activities in the experiments, the corrosion rate determined by the weight loss analyses increased and became closer to that predicted by the theoretical model.

These results highlight the limitations of assessing MIC impact solely based on cell abundance. The number of live cells under flow against static conditions is approximately 3-fold lower at 0.1 MPa and 2-fold lower at 30 MPa (Supplementary Table S3). This correlates with respective increases in corrosion rates by 8.7 and 4.1 times (Figure 2), leading to the hypothesis that MIC is not directly linked to the number of cells attached to the surface of a metal, but it is rather determined by the viability of the biofilm and its metabolic states (Little and Lee, 2015).

Thus, the observed differences in corrosion rates can primarily be attributed to two key factors: the continuous supply of fresh medium, as reported before by Taheri et al. (2005), and the concurrent removal of potentially toxic metabolic end-products, such as H_2_S (McCartney and Oleszkiewicz, 1991; Reis et al., 1992; Fatah et al., 2013), ultimately resulting in characteristic viabilities rates for each biofilms under different conditions.

Evidence of higher accumulation of H_2_S in the bulk at 0.1 MPa was reflected by the increased concentration of S in the corrosion product on the coupons incubated under static conditions (Table 1). It is well established that part of the sulphide generated by SRB reacts with the dissolved iron to form FeS and results in an easily recognizable black layer on the surface of the coupons (King and Miller, 1971; Hamilton, 1985).

The different concentrations of S in the corrosion products between static and flow conditions, along with the distinct levels of O between low and high hydrostatic pressure indicate that the composition of the corrosion products formed on the coupons may differ.

The characterization of the surface of the coupons incubated in static sterile medium (Figure 3) did not show visible effects related to the pressure in either static or flow sterile conditions although characteristic morphological features were observed in the corrosion products (Figure 5). This is in contrast with what had been previously reported (Zhang et al., 2009; Yang et al., 2017) but might be a result of the extremely low corrosion rate coupled with the limited duration of each incubation.

On the other hand, in static cultures of *P. profundus*, the coupon surfaces exhibited localized corrosion in the form of shallow pits (Figures 3E,F). This type of corrosion, commonly associated with SRB-induced MIC, may result from various factors, including a partial protective effect of the biofilm or disruption of the passive layer (Rao, 2012; Chen et al., 2014). LPR analyses under the same conditions indicated that the biofilm/corrosion product layer provided significant protection (Figure 6), suggesting that its uneven distribution may lead to higher corrosion rates in the exposed regions and consequent formation of pits.

In presence of flow, similar features on the corrosion product were visible but both pressures resulted in generalized corrosion with no distinguishable pits (Figures 3G–H).

Generalized uniform corrosion as a result of anaerobic MIC has not been described often in literature. We posit that it might be a characteristic of the early stage of the corrosion process in flow conditions related to the limited duration of the experiments and the slow metabolic rate of the bacteria.

The LPR measurements supported the corrosion rates measured through weight loss. Under sterile conditions, the initial low polarization resistance pointed to an initial corrosion phase, followed by the formation of a protective layer. This led to the increase in resistance and consequent decrease of corrosion rate, likely due to the deposition of corrosion products, which were instead partially removed by the flow.

In contrast, with one exception showing an initial increase followed by a trend comparable to the others, in *P. profundus* cultures, the LPR showed a general decline in the resistance starting from the onset of incubation. This phenomenon is commonly attributed to the progression of the corrosion process coupled with the formation of a conductive corrosion product layer, primarily composed of a mixture of Fe and FeS (AlAbbas et al., 2013). In most cases where corrosion is dominated by abiotic reactions, the corrosion rate decreases gradually with time as corrosion products adhere to the surface and form a diffusion barrier. In contrast to expectations based on a corrosive system where only the abiotic processes influence corrosion rate, microbes tend to increase the corrosion rate over time. Microbes may affect the base metal directly and cycle the elements of the corrosion products. Both biological and inorganic processes occur on metal surfaces in the presence of microbes within the same time period, but in the opposite direction at the metal-solution interface (Videla and Herrera, 2009). As seen in this study, the microorganisms were embedded in the corrosion-product layer and appeared to have a close interaction with the deposit layer.

The EIS analysis encountered significant limitations stemming from noise and the presence of multiple time-dependent RQ constants, likely due to the accumulation of biomass and corrosion products on the steel surface. This limitation was likely due to the complexity of the system, which will require developing a new type of connection between the electrodes and the potentiostat. Addressing this issue will be fundamental for future studies and routine analyses in high-pressure reactors. In this study, we used a single time constant equivalent circuit.

The $C_{eff}$ was much higher in the experiments inoculated with bacteria than in the abiotic experiments (Figure 7), indicating that the capacitance of the microbial EPS was higher than that of the inorganic corrosion products deposited on the steel surface in the abiotic corrosion process.

At low frequencies, the impedance calculated through one-time constant equivalent circuit (Supplementary Figure S2) simplifies to Z = R_e_ + R_t_. At the lowest frequency tested (0.01 Hz), the impedance Z was much higher in abiotic experiments than those inoculated with bacteria (Supplementary Figure S9), which indicates higher integrity of the metal surface under abiotic conditions (Martinez et al., 2024).

The higher $C_{eff}$ in the experiments at 30 MPa inoculated with bacteria under flow conditions (Supplementary Figure S7), coupled with the low impedance at low frequency (Supplementary Figure S3), could be considered as an indicator of a better protective effect of the biofilm formed under static conditions. Similar trend was observed for the $C_{eff}\;$ in abiotic experiments at 30 MPa (Figure 7), in which higher $C_{eff}\;$ and lower impedance were observed under flow conditions (Supplementary Figure S9), thereby indicating a better protective effect of the biofilm formed under static conditions.

The $C_{eff}\;$ for all the experiments increased with time, indicating the accumulation of corrosion products and biofilms. The increase of $C_{eff}\;$ in biotic experiment under flow was consistent with the formation of EPS-rich biofilm, which is commonly observed under flow conditions. The impedance at low frequency decreased initially for all experiments and then increased again in the abiotic experiments. However, in experiments inoculated with bacteria under flow conditions, the impedance did not stabilize, suggesting that there was an ongoing degradation of the metal surface, particularly under flow conditions. These observations are in agreement with previous literature on abiotic and microbially influenced corrosion (Moradi et al., 2022; Jiang et al., 2023; Martinez et al., 2024).

## 5 Conclusion

This study demonstrates the significant advantages of our newly designed continuous-flow high-pressure bio-electrochemostat over conventional experimental setups, such as static HPP or ambient pressure systems. Our findings emphasize the limitations of many existing studies, which often treat microorganisms merely as chemical entities, overlooking their viability and metabolic states. Here, we provide clear evidence that experimental setup modifications profoundly influence bacterial fitness and, consequently, their corrosive capabilities.

Additionally, the prevalent use of nutrient-rich media, rather than replicating the actual environmental availability of nutrients and chemicals, raises critical questions about the reliability of corrosion predictions under laboratory conditions.

The results also highlight that MIC in deep-sea environments might represent a threat to metallic structures comparable to that in shallow waters, emphasizing the need for more realistic experimental approaches. Recent studies have reported an increase in hydrogen embrittlement cracking attributed to sulfate-reducing bacteria under high hydrostatic pressure (Li et al., 2025). However, these experiments relied on compressed gas to simulate the pressure, which could introduce artifacts by increasing the partial pressure in the hydrogen gas phase. On the contrary, the use of our continuous-flow HHP bioreactor enables the investigation of complex microbial communities under more realistic deep-sea conditions, supported by extended incubation periods and enhanced electrochemical data quality.

The capability for real-time electrochemical measurements provided by this system offers a valuable tool for stakeholders to evaluate both existing and novel corrosion mitigation strategies. Moreover, the feasibility of incorporating controlled or modified conditions without the need for expensive and logistically challenging environmental experiments establishes a foundation for more cost-effective and sustainable research approaches in the field of MIC.

## Data Availability

The datasets presented in this study can be found in online repositories. The names of the repository/repositories and accession number(s) can be found at: https://github.com/NicoloIvanovich/Exploring-the-impact-of-flow-dynamics-in-deep-sea-corrosive-biofilms.

## References

[ref1] AlAbbas F. M. Williamson C. Bhola S. M. Spear J. R. Olson D. L. Mishra B.. (2013). Microbial corrosion in linepipe steel under the influence of a sulfate-reducing consortium isolated from an oil field. J. Mater. Eng. Perform. 22, 3517–3529. doi: 10.1007/s11665-013-0627-7

[ref2] Ashassi-Sorkhabi H. Moradi-Haghighi M. Zarrini G. (2012). The effect of Pseudoxanthomonas sp. as manganese oxidizing bacterium on the corrosion behavior of carbon steel. Mater. Sci. Eng. 32, 303–309. doi: 10.1016/j.msec.2011.10.033, PMID: 39909818

[ref3] ASTM (2107). Astm standard g1-03, practice for preparing, cleaning, and evaluating corrosion test specimens. West Conshohocken, PA: ASTM International.

[ref4] Audiffrin C. Cayol J.-L. Joulian C. Casalot L. Thomas P. Garcia J.-L.. (2003). *Desulfonauticus submarinus* gen. Nov., sp. nov., a novel sulfate-reducing bacterium isolated from a deep-sea hydrothermal vent. Int. J. Syst. Evol. Microbiol. 53, 1585–1590. doi: 10.1099/ijs.0.02551-0 13130052

[ref5] Bale S. J. Goodman K. Rochelle P. A. Marchesi J. R. Fry J. C. Weightman A. J.. (1997). *Desulfovibrio profundus* sp. nov., a novel barophilic sulfate-reducing bacterium from deep sediment layers in the Japan Sea. Int. J. Syst. Evol. Microbiol. 47, 515–521. doi: 10.1099/00207713-47-2-515, PMID: 9103642

[ref6] Beech I. B. Sztyler M. Gaylarde C. C. Smith W. L. Sunner J. (2014). “Biofilms and biocorrosion” in Understanding biocorrosion. eds. Liengen T. Féron D. Basséguy R. Beech I. B. (Oxford: Elsevier), 33–56.

[ref7] Bellou N. Papathanassiou E. Dobretsov S. Lykousis V. Colijn F. (2012). The effect of substratum type, orientation and depth on the development of bacterial deep-sea biofilm communities grown on artificial substrata deployed in the eastern Mediterranean. Biofouling 28, 199–213. doi: 10.1080/08927014.2012.662675, PMID: 22352335

[ref8] Brading M. Boyle J. Lappin-Scott H. (1995). Biofilm formation in laminar flow using *Pseudomonas fluorescens* EX101. J. Ind. Microbiol. Biotechnol. 15, 297–304. doi: 10.1007/BF01569983

[ref9] Chen Y. Howdyshell R. Howdyshell S. Ju L.-K. (2014). Characterizing pitting corrosion caused by a long-term starving sulfate-reducing bacterium surviving on carbon steel and effects of surface roughness. Corrosion 70, 767–780. doi: 10.5006/1190

[ref10] Chen H. Kimyon Ö. Ramandi H. L. Craig P. Gunawan C. Wu S.. (2022). Microbiologically influenced stress corrosion cracking responsible for catastrophic failure of cable bolts. Eng. Fail. Anal. 131:105884. doi: 10.1016/j.engfailanal.2021.105884

[ref11] Chen S. Qiu L. Sun S. Yang J. Meng Q. Yang W. (2021). Research progress on corrosion of equipment and materials in deep-sea environment. Adv. Civil Eng. 2021, 1–12. doi: 10.1155/2021/7803536, PMID: 39328480

[ref12] Chen L. Wei B. Xu X. (2021). Effect of sulfate-reducing bacteria (SRB) on the corrosion of buried pipe steel in acidic soil solution. Coatings 11:625. doi: 10.3390/coatings11060625

[ref13] Chohan I. M. Ahmad A. Sallih N. Bheel N. Salilew W. M. Almaliki A. H. (2024). Effect of seawater salinity, pH, and temperature on external corrosion behavior and microhardness of offshore oil and gas pipeline: RSM modelling and optimization. Sci. Rep. 14:16543. doi: 10.1038/s41598-024-67463-2, PMID: 39019941 PMC11255295

[ref14] Coulson J. M. Richardson J. F. Backhurst J. R. Harker J. H. (1990). Chemical engineering: Fluid flow, heat transfer and mass transfer. Oxford: Pergamon press.

[ref15] Dang Y. T. H. Power A. Cozzolino D. Dinh K. B. Ha B. S. Kolobaric A.. (2022). Analytical characterisation of material corrosion by biofilms. J. Bio Tribo Corrosion 8:50. doi: 10.1007/s40735-022-00648-2

[ref16] Dominguez-Benetton X. Sevda S. Vanbroekhoven K. Pant D. (2012). The accurate use of impedance analysis for the study of microbial electrochemical systems. Chem. Soc. Rev. 41, 7228–7246. doi: 10.1039/C2CS35026B, PMID: 22885371

[ref17] Dong Y. Jiang B. Xu D. Jiang C. Li Q. Gu T. (2018). Severe microbiologically influenced corrosion of S32654 super austenitic stainless steel by acid producing bacterium *Acidithiobacillus caldus* SM-1. Bioelectrochemistry 123, 34–44. doi: 10.1016/j.bioelechem.2018.04.014, PMID: 29723805

[ref18] Dou W. Liu J. Cai W. Wang D. Jia R. Chen S.. (2019). Electrochemical investigation of increased carbon steel corrosion via extracellular electron transfer by a sulfate reducing bacterium under carbon source starvation. Corros. Sci. 150, 258–267. doi: 10.1016/j.corsci.2019.02.005

[ref19] Enning D. Venzlaff H. Garrelfs J. Dinh H. T. Meyer V. Mayrhofer K.. (2012). Marine sulfate-reducing bacteria cause serious corrosion of iron under electroconductive biogenic mineral crust. Environ. Microbiol. 14, 1772–1787. doi: 10.1111/j.1462-2920.2012.02778.x, PMID: 22616633 PMC3429863

[ref20] Fang J. Zhang L. Bazylinski D. A. (2010). Deep-sea piezosphere and piezophiles: geomicrobiology and biogeochemistry. Trends Microbiol. 18, 413–422. doi: 10.1016/j.tim.2010.06.006, PMID: 20663673

[ref21] Fatah M. C. Ismail M. C. Wahjoedi B. A. (2013). Effects of sulphide ion on corrosion behaviour of X52 steel in simulated solution containing metabolic products species: a study pertaining to microbiologically influenced corrosion (MIC). Corros. Eng. Sci. Technol. 48, 211–220. doi: 10.1179/1743278212Y.0000000065

[ref22] Fick A. (1855). V. On liquid diffusion. The London, Edinburgh, and Dublin philosophical magazine. J. Sci. 10, 30–39. doi: 10.1080/14786445508641925, PMID: 39845729

[ref23] Foustoukos D. I. Pérez-Rodríguez I. (2015). A continuous culture system for assessing microbial activities in the piezosphere. Appl. Environ. Microbiol. 81, 6850–6856. doi: 10.1128/AEM.01215-15, PMID: 26209666 PMC4561707

[ref24] Gage J. D. Tyler P. A. (1991). Deep-Sea biology: A natural history of organisms at the Deep-Sea floor. Cambridge: Cambridge University Press, 9–30.

[ref25] Guan Y. Hikmawan T. Antunes A. Ngugi D. Stingl U. (2015). Diversity of methanogens and sulfate-reducing bacteria in the interfaces of five deep-sea anoxic brines of the Red Sea. Res. Microbiol. 166, 688–699. doi: 10.1016/j.resmic.2015.07.002, PMID: 26192212

[ref26] Guan F. Liu Z. Dong X. Zhai X. Zhang B. Duan J.. (2021). Synergistic effect of carbon starvation and exogenous redox mediators on corrosion of X70 pipeline steel induced by Desulfovibrio singaporenus. Sci. Total Environ. 788:147573. doi: 10.1016/j.scitotenv.2021.147573, PMID: 34034174

[ref27] Guezennec J. Ortega-Morales O. Raguenes G. Geesey G. (1998). Bacterial colonization of artificial substrate in the vicinity of deep-sea hydrothermal vents. FEMS Microbiol. Ecol. 26, 89–99. doi: 10.1111/j.1574-6941.1998.tb00495.x

[ref28] Hamdan L. J. Hampel J. J. Moseley R. D. Mugge R. L. Ray A. Salerno J. L.. (2021). Deep-sea shipwrecks represent island-like ecosystems for marine microbiomes. ISME J. 15, 2883–2891. doi: 10.1038/s41396-021-00978-y, PMID: 33888864 PMC8443566

[ref29] Hamilton W. A. (1985). Sulphate-reducing bacteria and anaerobic corrosion. Ann. Rev. Microbiol. 39, 195–217. doi: 10.1146/annurev.mi.39.100185.001211, PMID: 3904600

[ref30] Heitz E. Flemming H. C. Sand W. (1996). Microbially influenced corrosion of materials: Scientific and engineering aspects. Berlin: Springer.

[ref31] Houghton J. L. Seyfried W. E. Banta A. B. Reysenbach A. L. (2007). Continuous enrichment culturing of thermophiles under sulfate and nitrate-reducing conditions and at deep-sea hydrostatic pressures. Extremophiles 11, 371–382. doi: 10.1007/s00792-006-0049-7, PMID: 17221162

[ref32] Hubert C. Nemati M. Jenneman G. Voordouw G. (2005). Corrosion risk associated with microbial souring control using nitrate or nitrite. Appl. Microbiol. Biotechnol. 68, 272–282. doi: 10.1007/s00253-005-1897-2, PMID: 15711941

[ref33] Incropera F. P. DeWitt D. P. Bergman T. L. Lavine A. S. (1996). Fundamentals of heat and mass transfer. New York: Wiley.

[ref34] ITCC (2011). Fresh water and seawater properties. *Reccomended procedures and guidelines* 7.5-02-01-03.

[ref35] Jannasch H. W. Wirsen C. O. Doherty K. W. (1996). A pressurized chemostat for the study of marine barophilic and oligotrophic bacteria. Appl. Environ. Microbiol. 62, 1593–1596. doi: 10.1128/AEM.62.5.1593-1596.1996, PMID: 16535311 PMC1388849

[ref36] Javed M. A. Ivanovich N. Messinese E. Liu R. Astorga S. E. Yeo Y. P.. (2024). The role of metallurgical features in the Microbially influenced corrosion of carbon steel: a critical review. Microorganisms 12:892. doi: 10.3390/microorganisms12050892, PMID: 38792722 PMC11124232

[ref37] Jiang Y. Bustillo K. C. Devine T. M. (2023). Investigation via electron microscopy and electrochemical impedance spectroscopy of the effect of aqueous zinc ions on passivity and the surface films of alloy 600 in PWR PW at 320°C. Corrosion Materials Degradation 4, 54–89. doi: 10.3390/cmd4010005

[ref38] Khelaifia S. Fardeau M.-L. Pradel N. Aussignargues C. Garel M. Tamburini C.. (2011). *Desulfovibrio piezophilus* sp. nov., a piezophilic, sulfate-reducing bacterium isolated from wood falls in the Mediterranean Sea. Int. J. Syst. Evol. Microbiol. 61, 2706–2711. doi: 10.1099/ijs.0.028670-0, PMID: 21169465

[ref39] King R. A. Miller J. D. A. (1971). Corrosion by sulphate-reducing bacteria. Nature 233, 491–492. doi: 10.1038/233491a0, PMID: 16063454

[ref40] Krsmanovic M. Biswas D. Ali H. Kumar A. Ghosh R. Dickerson A. K. (2021). Hydrodynamics and surface properties influence biofilm proliferation. Adv. Colloid Interf. Sci. 288:102336. doi: 10.1016/j.cis.2020.102336, PMID: 33421727

[ref41] Kunes J. (2012). Dimensionless physical quantities in science and engineering. Amsterdam: Elsevier.

[ref42] Lahme S. Enning D. Callbeck C. M. Vega D. M. Curtis T. P. Head I. M.. (2019). Metabolites of an oil field sulfide-oxidizing, nitrate-reducing Sulfurimonas sp. cause severe corrosion. Appl. Environ. Microbiol. 85, e01891–e01818. doi: 10.1128/AEM.01891-18, PMID: 30446554 PMC6344618

[ref43] Lanneluc I. Langumier M. Sabot R. Jeannin M. Refait P. Sablé S. (2015). On the bacterial communities associated with the corrosion product layer during the early stages of marine corrosion of carbon steel. Int. Biodeterior. Biodegradation 99, 55–65. doi: 10.1016/j.ibiod.2015.01.003

[ref44] Li J. Zhou E. Xie F. Li Z. Wang F. Xu D. (2025). Accelerated stress corrosion cracking of X80 pipeline steel under the combined effects of sulfate-reducing bacteria and hydrostatic pressure. Corros. Sci. 243:112593. doi: 10.1016/j.corsci.2024.112593

[ref45] Little B. J. Blackwood D. J. Hinks J. Lauro F. M. Marsili E. Okamoto A.. (2020). Microbially influenced corrosion—any progress? Corros. Sci. 170:108641. doi: 10.1016/j.corsci.2020.108641

[ref46] Little B. J. Lee J. S. (2015). Microbiologically influenced corrosion. Oil Gas Pipelines, 387–398. doi: 10.1002/9781119019213.ch27

[ref47] Liu R. Liu L. Wang F. (2022). The role of hydrostatic pressure on the metal corrosion in simulated deep-sea environments — a review. J. Materials Sci. Technol. 112, 230–238. doi: 10.1016/j.jmst.2021.10.014

[ref48] Lv M. Chen X. Li Z. Du M. (2021). Effect of sulfate-reducing bacteria on hydrogen permeation and stress corrosion cracking behavior of 980 high-strength steel in seawater. J. Materials Sci. Technol. 92, 109–119. doi: 10.1016/j.jmst.2021.02.039

[ref49] Ma R. Shen Y. Wang C. Dong J. Ke W. (2022). Effect of hydrostatic pressure on the thermodynamic and kinetic behavior of metal electrode reactions. Electrochim. Acta 424:140617. doi: 10.1016/j.electacta.2022.140617

[ref50] Martinez S. Hudec B. Šoić I. (2024). Low-frequency EIS interpretation with the potential to predict the durability of protective coatings. Prog. Org. Coat. 197:108811. doi: 10.1016/j.porgcoat.2024.108811

[ref51] McCartney D. M. Oleszkiewicz J. A. (1991). Sulfide inhibition of anaerobic degradation of lactate and acetate. Water Res. 25, 203–209. doi: 10.1016/0043-1354(91)90030-T

[ref52] Moradi M. Ghiara G. Spotorno R. Xu D. Cristiani P. (2022). Understanding biofilm impact on electrochemical impedance spectroscopy analyses in microbial corrosion and microbial corrosion inhibition phenomena. Electrochim. Acta 426:140803. doi: 10.1016/j.electacta.2022.140803

[ref53] Moseley R. D. Hampel J. J. Mugge R. L. Hamdan L. J. (2022). Historic wooden shipwrecks influence dispersal of deep-sea biofilms. Front. Mar. Sci. 9:873445. doi: 10.3389/fmars.2022.873445

[ref54] Mugge R. L. Brock M. L. Salerno J. L. Damour M. Church R. A. Lee J. S.. (2019). Deep-Sea biofilms, historic shipwreck preservation and the Deepwater horizon spill. Front. Mar. Sci. 6:48. doi: 10.3389/fmars.2019.00048

[ref55] Murthy P. S. Mohan T. V. K. Nanchariah Y. V. Adhikari S. Ramadass G. A. Gupta G. V. M.. (2023). “Biodiversity of deep ocean on development of biofilms: biofouling communities and corrosion performance of materials” in Advances in nanotechnology for marine antifouling (Elsevier), Amsterdam, 141–164.

[ref56] NACE (2024). Assessment of the global cost of corrosion. Available online at: http://impact.nace.org/economic-impact.aspx (Accessed January 3, 2025)

[ref57] Newton I. (1701). Scala graduum caloris. Philos. Transac. Royal Soc. 22, 824–829.

[ref58] Pal M. K. Lavanya M. (2022). Microbial influenced corrosion: understanding bioadhesion and biofilm formation. J. Bio Tribo Corrosion 8:76. doi: 10.1007/s40735-022-00677-x

[ref59] Parsekar A. S. Jobby R. (2023). “Deep-sea extremophiles and their diversity in the Indian Ocean” in Extremophiles. eds. Maulin P. S. Satarupa D. (Berlin, Boston: De Gruyter), 65–94.

[ref60] Pradel N. Ji B. Gimenez G. Talla E. Lenoble P. Garel M.. (2013). The first genomic and proteomic characterization of a deep-sea sulfate reducer: insights into the piezophilic lifestyle of *Desulfovibrio piezophilus*. PLoS One 8:e55130. doi: 10.1371/journal.pone.0055130, PMID: 23383081 PMC3559428

[ref61] Procópio L. (2019). The role of biofilms in the corrosion of steel in marine environments. World J. Microbiol. Biotechnol. 35:73. doi: 10.1007/s11274-019-2647-4, PMID: 31037431

[ref62] Qian H. Liu S. Wang P. Huang Y. Lou Y. Huang L.. (2020). Investigation of microbiologically influenced corrosion of 304 stainless steel by aerobic thermoacidophilic archaeon *Metallosphaera cuprina*. Bioelectrochemistry 136:107635. doi: 10.1016/j.bioelechem.2020.107635, PMID: 32866835

[ref63] Rajala P. Bomberg M. Vepsäläinen M. Carpén L. (2017). Microbial fouling and corrosion of carbon steel in deep anoxic alkaline groundwater. Biofouling 33, 195–209. doi: 10.1080/08927014.2017.1285914, PMID: 28198664

[ref64] Rajala P. Cheng D.-Q. Rice S. A. Lauro F. M. (2022). Sulfate-dependant microbially induced corrosion of mild steel in the deep sea: a 10-year microbiome study. Microbiome 10:4. doi: 10.1186/s40168-021-01196-6, PMID: 35027090 PMC8756651

[ref65] Rao T. S. (2012). “Microbial fouling and corrosion: fundamentals and mechanisms” in Operational and environmental consequences of large industrial cooling water systems. eds. Rajagopal S. Jenner H. Venugopalan V. (Boston, MA: Springer US), 95–126.

[ref66] Reis M. A. M. Almeida J. S. Lemos P. C. Carrondo M. J. T. (1992). Effect of hydrogen sulfide on growth of sulfate reducing bacteria. Biotechnol. Bioeng. 40, 593–600. doi: 10.1002/bit.260400506, PMID: 18601155

[ref67] Rempel C. L. Evitts R. W. Nemati M. (2006). Dynamics of corrosion rates associated with nitrite or nitrate mediated control of souring under biological conditions simulating an oil reservoir. J. Ind. Microbiol. Biotechnol. 33, 878–886. doi: 10.1007/s10295-006-0142-z, PMID: 16758172

[ref68] Taheri R. A. Nouhi A. Hamedi J. Javaherdashti R. (2005). Comparison of corrosion rates of some steels in batch and semi-continuous cultures of sulfate-reducing bacteria. Asian J. Microbiol. Biotechnol. Environ. Sci. 7:5.

[ref69] Venkatesan R. Dwarakadasa E. S. Ravindran M. (2003). Biofilm formation on structural materials in deep sea environments. New Delhi: NISCAIR-CSIR.

[ref70] Videla H. A. Herrera L. K. (2009). Understanding microbial inhibition of corrosion. A comprehensive overview. Int. Biodeterior. Biodegradation 63, 896–900. doi: 10.1016/j.ibiod.2009.02.002

[ref71] Videla H. A. Herrera L. K. Edyvean G. (2005). An updated overview of SRB induced corrosion and protection of carbon steel. Corrosion.

[ref72] Wang D. Kijkla P. Mohamed M. E. Saleh M. A. Kumseranee S. Punpruk S.. (2021). Aggressive corrosion of carbon steel by Desulfovibrio ferrophilus IS5 biofilm was further accelerated by riboflavin. Bioelectrochemistry 142:107920. doi: 10.1016/j.bioelechem.2021.107920, PMID: 34388603

[ref73] Wang D. Zhou E. Xu D. Lovley D. R. (2023). Burning question: are there sustainable strategies to prevent microbial metal corrosion? Microb. Biotechnol. 16, 2026–2035. doi: 10.1111/1751-7915.14347, PMID: 37796110 PMC10616648

[ref74] Wirsen C. O. Molyneaux S. J. (1999). A study of deep-sea natural microbial populations and barophilic pure cultures using a high-pressure chemostat. Appl. Environ. Microbiol. 65, 5314–5321. doi: 10.1128/aem.65.12.5314-5321.1999, PMID: 10583982 PMC91722

[ref75] Wood J. L. Neil W. C. Wade S. A. (2021). High taxonomic diversity in ship bilges presents challenges for monitoring microbial corrosion and opportunity to utilize community functional profiling. Appl. Environ. Microbiol. 87, e0089021–e0000821. doi: 10.1128/AEM.00890-21, PMID: 34232755 PMC8388792

[ref76] Wu T. Yan M. Yu L. Zhao H. Sun C. Yin F.. (2019). Stress corrosion of pipeline steel under disbonded coating in a SRB-containing environment. Corros. Sci. 157, 518–530. doi: 10.1016/j.corsci.2019.06.026

[ref77] Wu Y. Zhao W. Wang L. (2025). State of the art and current trends on the metal corrosion and protection strategies in deep sea. J. Materials Sci. Technol. 215, 192–213. doi: 10.1016/j.jmst.2024.07.026

[ref78] Xu D. Gu T. (2014). Carbon source starvation triggered more aggressive corrosion against carbon steel by the *Desulfovibrio vulgaris* biofilm. Int. Biodeterior. Biodegradation 91, 74–81. doi: 10.1016/j.ibiod.2014.03.014

[ref79] Yang Z. Kan B. Li J. Qiao L. Volinsky A. A. Su Y. (2017). A statistical study on the effect of hydrostatic pressure on metastable pitting corrosion of X70 pipeline steel. Materials (Basel) 10:1307. doi: 10.3390/ma10111307, PMID: 29135912 PMC5706254

[ref80] Yang J. Wang Z. B. Qiao Y. X. Zheng Y. G. (2022). Synergistic effects of deposits and sulfate reducing bacteria on the corrosion of carbon steel. Corros. Sci. 199:110210. doi: 10.1016/j.corsci.2022.110210

[ref81] Yayanos A. A. (1986). Evolutional and ecological implications of the properties of deep-sea barophilic bacteria. Proc. Natl. Acad. Sci. 83, 9542–9546. doi: 10.1073/pnas.83.24.9542, PMID: 16593790 PMC387176

[ref82] Zhang T. Yang Y. Shao Y. Meng G. Wang F. (2009). A stochastic analysis of the effect of hydrostatic pressure on the pit corrosion of Fe–20Cr alloy. Electrochim. Acta 54, 3915–3922. doi: 10.1016/j.electacta.2009.02.010

[ref83] Zhang C. Zhang Z.-W. Liu L. (2016). Degradation in pitting resistance of 316L stainless steel under hydrostatic pressure. Electrochim. Acta 210, 401–406. doi: 10.1016/j.electacta.2016.05.169

[ref84] Zheng R. Wu S. Sun C. (2021). Pseudodesulfovibrio cashew sp. nov., a novel deep-sea sulfate-reducing bacterium, linking heavy metal resistance and sulfur cycle. Microorganisms 9:429. doi: 10.3390/microorganisms9020429, PMID: 33669756 PMC7922080

