# Supplementary material for: Exploring the impact of flow dynamics on corrosive biofilms under simulated deep-sea high-pressure conditions using bio-electrochemostasis
Source: Front Microbiol. 2025 Feb 28;16:1540664. doi: 10.3389/fmicb.2025.1540664 (PMC11908379; doi:10.3389/fmicb.2025.1540664)
Supplement: Supplementary file 1 [file Data_Sheet_1.pdf]

Supplementary material

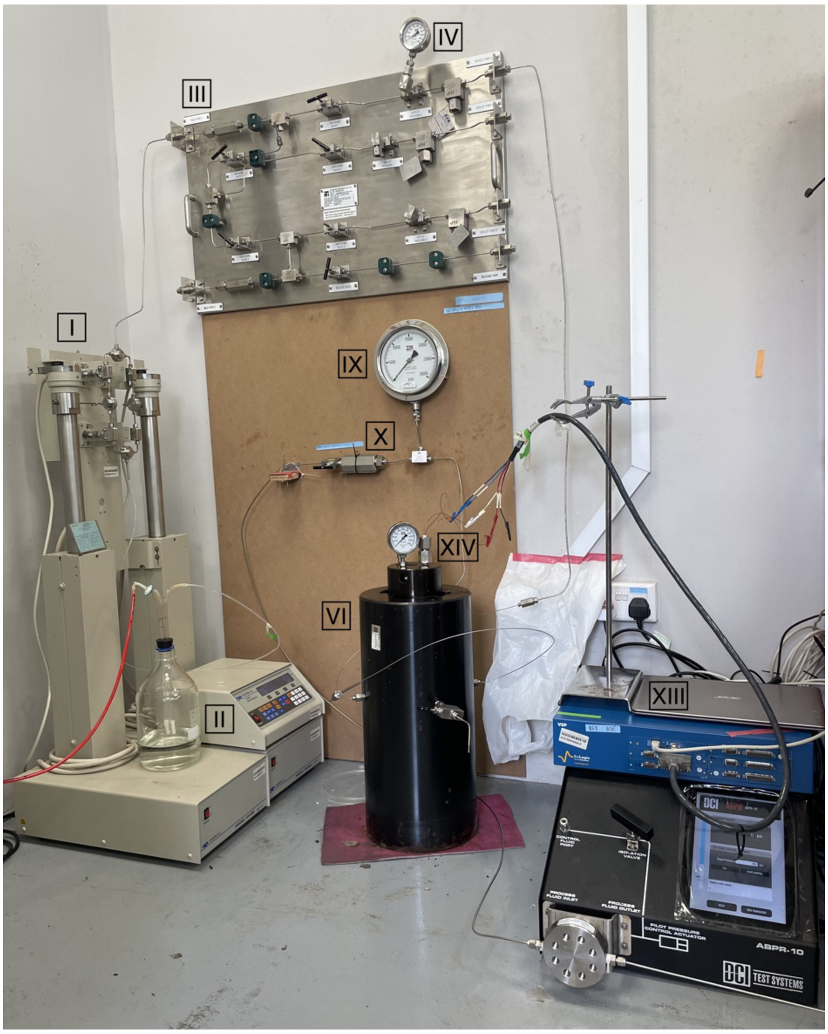

Fig. S1. Photo of the high-pressure bioelectrochemostat. Visible are the dual pump continuous-flow system (**I**), anaerobic medium reservoir (II), control panel (**III**), pre-reactor pressure gauge (**IV**), pressure vessel (**VI**), post-reactor pressure gauge (**IX**), flow check valve (**X**), ABPR (**XI**), high-pressure connector (**XIV**) and potentiostat (**XIII**).

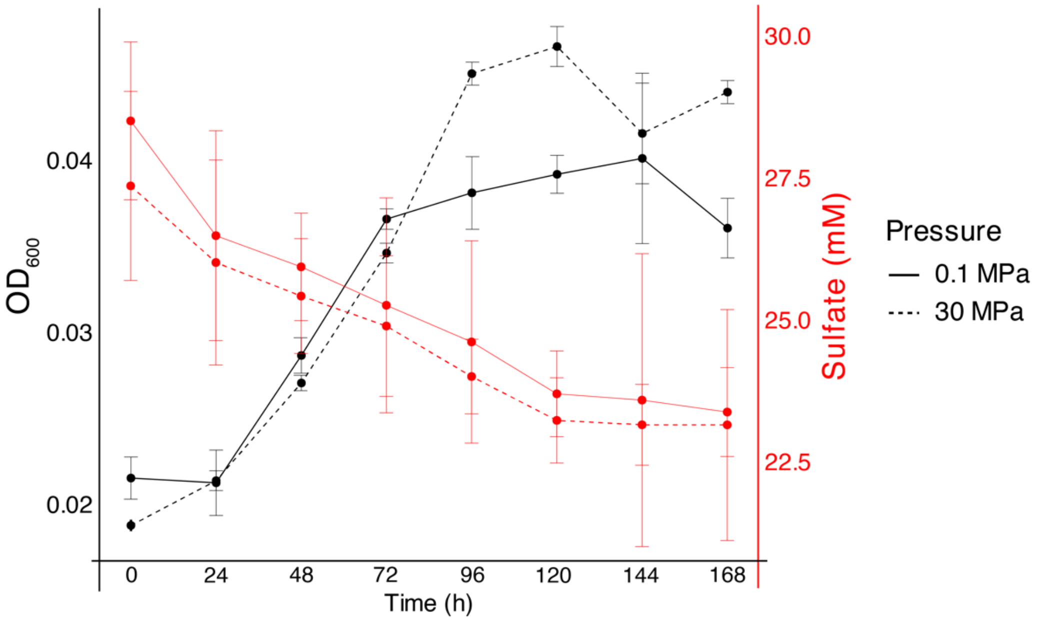

Fig. S2. Growth rate and sulfate consumption of *P. profundus* in ASW at 0.1 and 30 MPa ~~was~~ measured using the same preparation adopted for the experiments. The error bars indicate the standard deviation calculated on the 4 replicates.

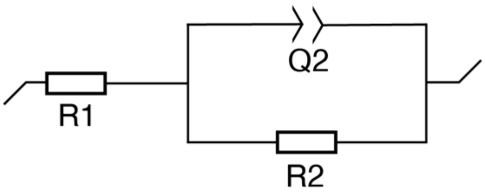

Fig. S3. EIS results were fitted using a single constant equivalent circuit. Where R1 corresponds to the resistance of the electrolyte, R2 represents the charge transfer resistance (Rt), and Q2 is the Constant Phase Element (CPE).

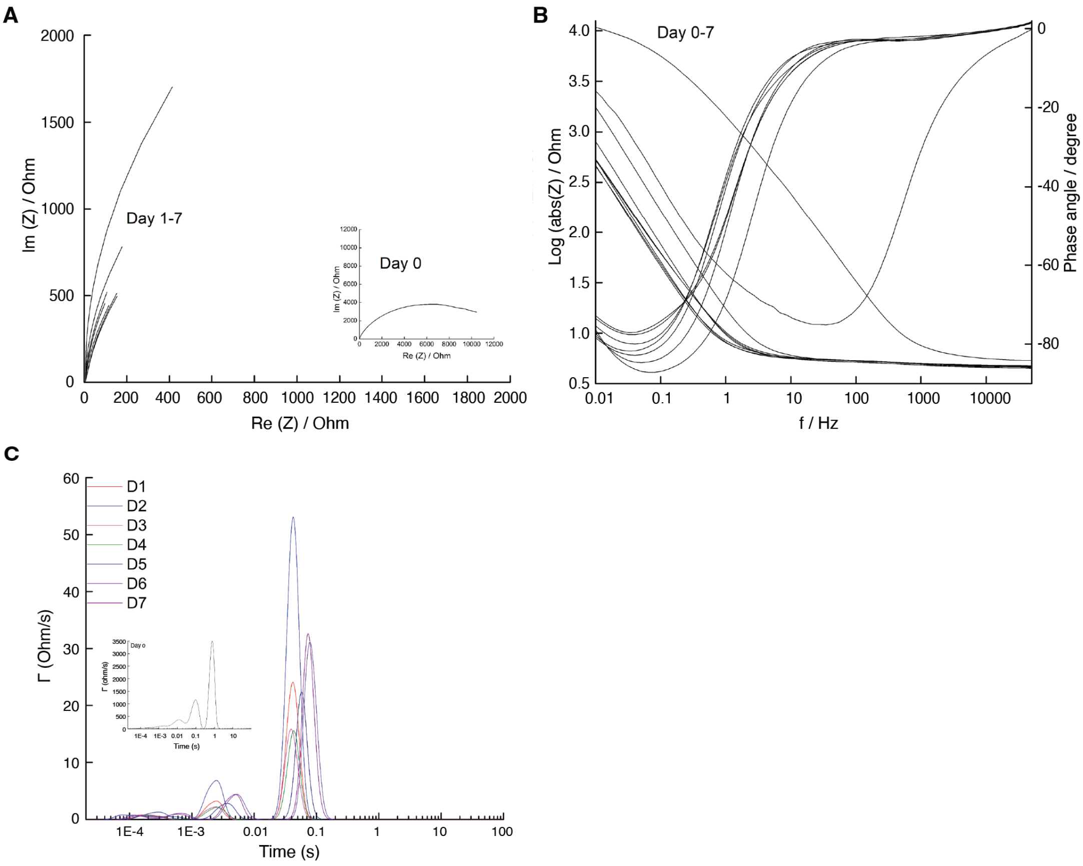

Figure S4. Representative set of EIS plots including Nyquist plot (**A**), Bode plot (**B**) and distribution function of relaxation times (DFRT) plot (**C**). With the exception of the sample immediately after inoculation (D0), which shows multiple time constants, all other samples are adequately described with a one-time constant model. The dataset shown corresponds to one of the biological replicate at 30 MPa under static conditions.

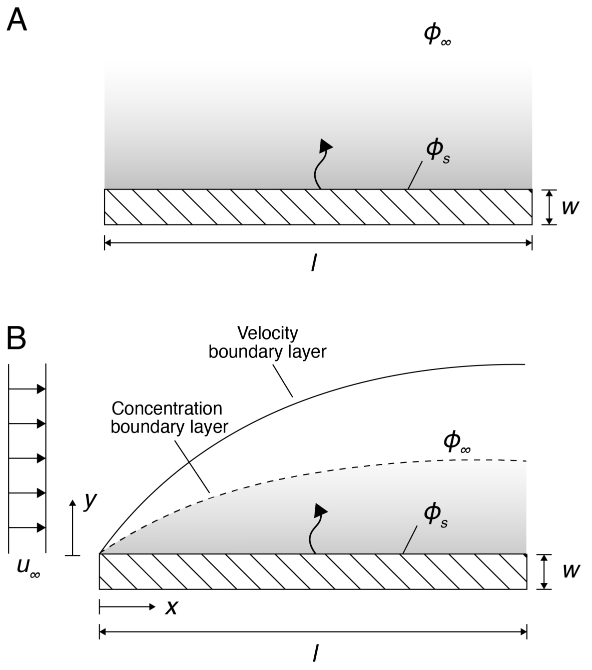

Fig. S5. Schematics of the corrosion model of the coupon. **A** Without the background flow, the substance diffuses from the plate surface of species concentration, φ_s, to the bulk fluid of species concentration, φ_∞. **B** In the continuous flow condition with the averaged flow velocity of u_∞, the mass transfer rate of the corrosion products released from the coupon's surface to the bulk fluid is speed up by convection. In the experimental setup, the concentration boundary layer of the corrosion products is well contained in the velocity boundary layer of the flow.

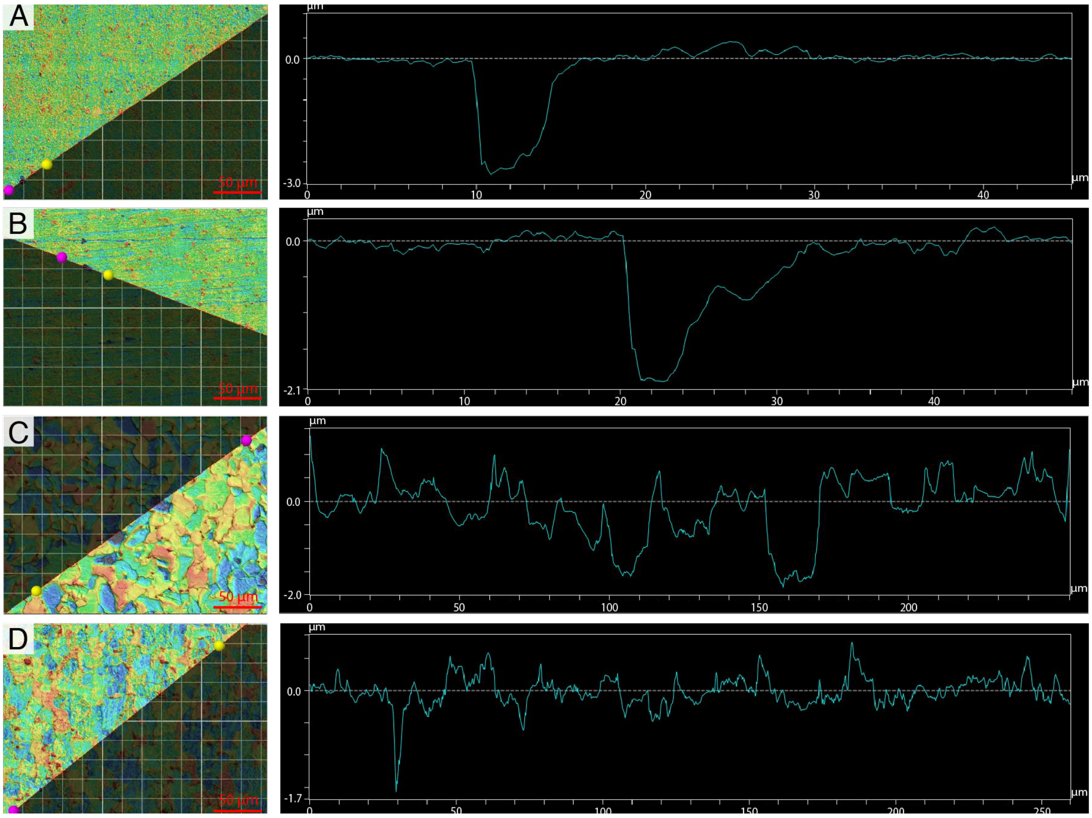

Fig. S6. Maximum pit depths on the surface of coupons incubated with *P. profundus* were obtained through CLSM analysis. **A, B** refer to 0.1 and 30 MPa in static conditions. **C, D** to constant flow at 0.1 and 30 MPa.

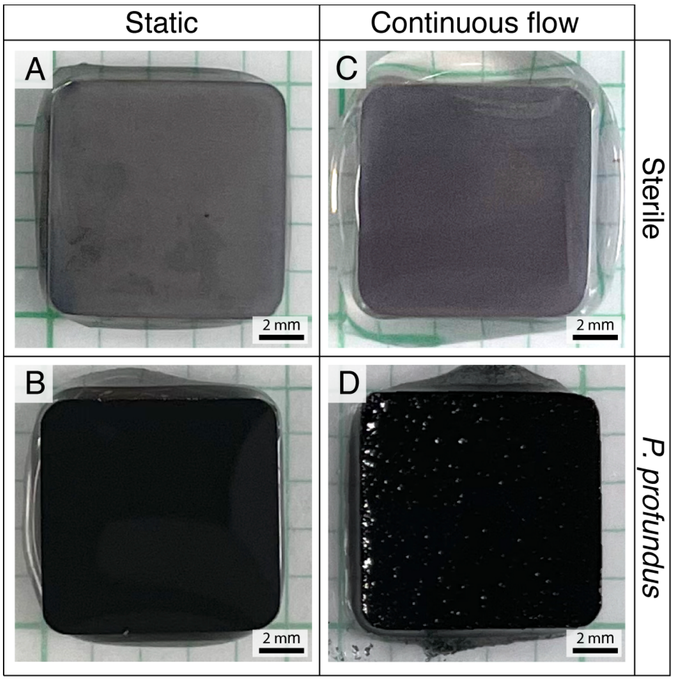

Fig. S7. Aspects of the coupons after incubation at 30 MPa under static (**A, B**) and continuous flow conditions (**C, D**) in abiotic (**A, C**) and P. profundus cultures (**B, D**).

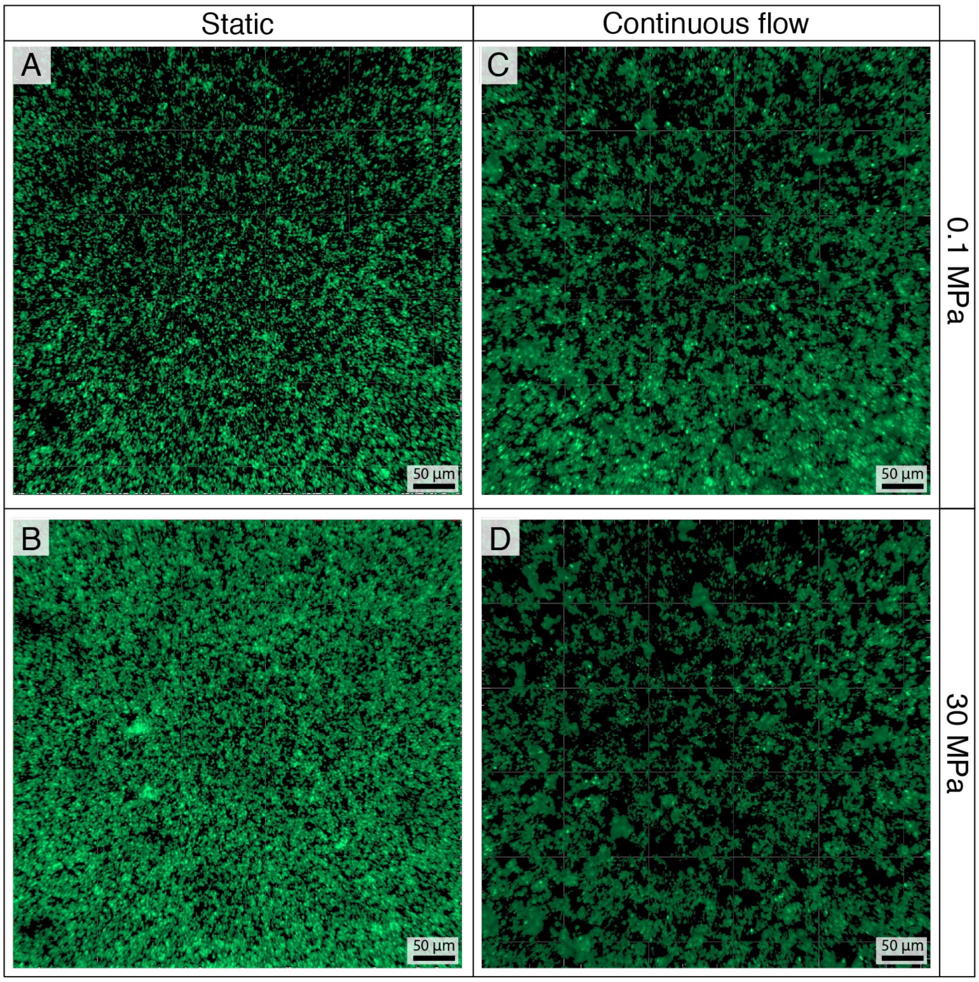

Fig. S8. *P. profundus* biofilm matrix was characterised after staining with Syto9 under CLSM in static (**A, B**) and continuous flow conditions (**C, D**).

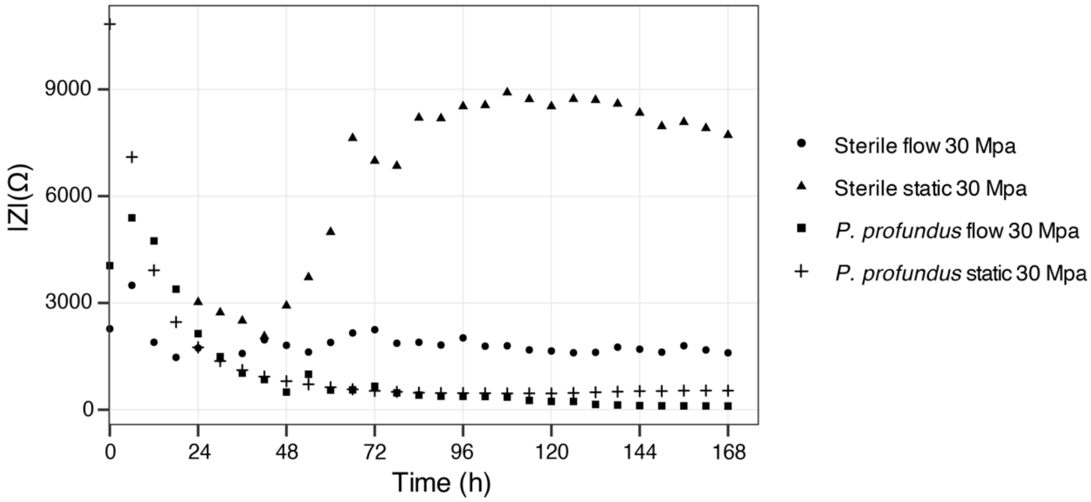

Fig. S9. Impedance (Z) calculated at 0.01 Hz throughout the entire duration of the experiment.

	C	Si	Mn	P	S	Cu
wt%	0.11	0.23	0.135	0.011	0.002	0.01
	**Ni**	**Cr**	**Mo**	**Nb**	**V**	**Fe**
wt%	0.01	0.02	0.01	0.01	0.02	balance

Table S1: Chemical composition (in wt%) of the metal used for the experiments, as stated in inspection certificate provided by supplier (Nippon Steel Corp., Japan).

	**Static**		**Continuous flow**	
**Sterile medium**	0.1 MPa	30 MPa	0.1 MPa	30 MPa
P. profundus	0.1 MPa	30 MPa	0.1 MPa	30 MPa

Table S2. The effect of the flow in *P. profundus* cultures and sterile medium was analysed at atmospheric pressure (0.1 MPa) and HHP (30 MPa).

	**Static**		
	Live	Dead	Total
0.1 MPa	190±59.4	3170±127.28	3360±140.46
30MPa	116±22.63	2200±248.90	2316±249.39
	**Continuous flow**		
	Live	Dead	Total
0.1 MPa	576±175.6	238±82.02	814±193.6
30MPa	292±73.54	152±45.25	444±86.35

Table S3. Biofilm density in cells mm^{-2} and cells viability were characterised under CLSM after Syto9 and Propidium Iodide staining.

	**Sterile medium**				***P. profundus***			
	Static		Continuous flow		Static		Continuous flow	
	0.1 MPa	30 MPa	0.1 MPa	30 MPa	0.1 MPa	30 MPa	0.1 MPa	30 MPa
0 h	2698± 480	4686± 321	4055± 454	2651± 183	6605.5± 1032.5	6965.5± 874.5	4340± 65	5144± 739
24 h	4612.5± 1808.5	4394± 744	2881± 236	2677.5± 1622.5	7191± 1052	5141± 172	3874± 2070	2095.5± 291.5
48 h	4702± 1676	4340± 1134	6124± 2393	3028± 1929	4678.5± 57535	4064.5± 51.5	2789± 2065	1454.5± 730.5
72 h	5345.5± 1956.5	7394.5± 1284.5	8549.5± 2339.5	8467± 63	4737± 57	3623.5± 127.5	2891.5± 1536.5	1764± 399
96 h	9338± 159	9203± 1226	7927± 1467	6219± 429	2975.5± 1003.5	3246.5± 119.5	2894± 1085	1726± 83
120 h	11471± 171.5	9503± 791	8545.5± 64.5	6321± 51	1523.5± 1067.5	2982.5± 416.5	1500.5± 90.5	1180± 230
144 h	12267.5± 137.5	9010.5± 128.5	8537± 777	6018.5± 178.5	2744.5± 441.5	2895± 88	1333± 53	1197.5± 82.5
168 h	9687.5± 1073.5	9387± 583	8517± 627	6287± 157	2372± 325	2705± 30	1461.5± 45.5	1165± 251

Table S4. R_p values determined as the slope of the I/E curve in the proximity of the E_{corr}.
